# Hidden layers of human small RNAs

**DOI:** 10.1186/1471-2164-9-157

**Published:** 2008-04-10

**Authors:** Hideya Kawaji, Mari Nakamura, Yukari Takahashi, Albin Sandelin, Shintaro Katayama, Shiro Fukuda, Carsten O Daub, Chikatoshi Kai, Jun Kawai, Jun Yasuda, Piero Carninci, Yoshihide Hayashizaki

**Affiliations:** 1 Functional RNA Research Program, RIKEN Frontier Research System, 2-1 Hirosawa, Wako, Saitama 351-0198, Japan; 2 Genome Exploration Research Group, RIKEN Genomic Sciences Center(GSC), RIKEN Yokohama Institute, 1-7-22 Suehiro-cho, Tsurumi-ku, Yokohama, Kanagawa, 230-0045, Japan; 3 NTT Software Corporation, Teisan Kannai Bldg. 209, Yamashita-cho Naka-ku, Yokohama, Kanagawa, 231-8551, Japan; 4 The Bioinformatics Centre, Department of Biology & Biotech Research and Innovation Centre, University of Copenhagen, Ole Maaløes Vej 5, DK-2100 København ∅, Denmark; 5 Genome Science Laboratory, Discovery and Research Institute, RIKEN Wako Main Campus, 2-1 Hirosawa, Wako, Saitama 351-0198, Japan

## Abstract

**Background:**

Small RNA attracts increasing interest based on the discovery of RNA silencing and the rapid progress of our understanding of these phenomena. Although recent studies suggest the possible existence of yet undiscovered types of small RNAs in higher organisms, many studies to profile small RNA have focused on miRNA and/or siRNA rather than on the exploration of additional classes of RNAs.

**Results:**

Here, we explored human small RNAs by unbiased sequencing of RNAs with sizes of 19–40 nt. We provide substantial evidences for the existence of independent classes of small RNAs. Our data shows that well-characterized non-coding RNA, such as tRNA, snoRNA, and snRNA are cleaved at sites specific to the class of ncRNA. In particular, tRNA cleavage is regulated depending on tRNA type and tissue expression. We also found small RNAs mapped to genomic regions that are transcribed in both directions by bidirectional promoters, indicating that the small RNAs are a product of dsRNA formation and their subsequent cleavage. Their partial similarity with ribosomal RNAs (rRNAs) suggests unrevealed functions of ribosomal DNA or interstitial rRNA. Further examination revealed six novel miRNAs.

**Conclusion:**

Our results underscore the complexity of the small RNA world and the biogenesis of small RNAs.

## Background

Upon the discovery of the RNAi phenomena in invertebrates [1] and the discovery of functional siRNAs in mammalian cell systems [2,3], the identification of small RNAs has become an important area of research. Contrary to initial expectations, early studies based on the isolation and sequencing of small RNAs (20–25 nt) revealed the existence of a class of highly expressed small RNAs termed micro RNAs (miRNAs). Since then, miRNAs have been shown to regulate mRNA expression in a large variety of biological contexts, including cell development, differentiation and cancer. Recent studies have discovered further miRNAs by large-scale sequencing based on recent advances in sequencing technology [4-8].

Other efforts to isolate small RNAs (< 50~500 nt in size), sometimes termed "(experimental) RNomics" [9-11], have systematically identified additional species of small RNAs. Recently, the analysis of RNAs binding to the piwi family proteins has revealed a novel, small (29–30 nt), and yet uncharacterized class of RNAs restricted to the germ line cells [12-15], which does not depend on cleavage by Dicer [16,17]. These discoveries suggest the possibility of yet undiscovered classes of small RNAs in mammals. Despite this, recent studies to profile small RNAs have focused on miRNA and/or siRNA rather than on the exploration of additional classes of RNAs [4-6,8].

Here, we explore small RNAs by large-scale unbiased sequencing of RNAs with sizes of 20–40 nt. We provide substantial evidences for independent classes of small RNAs. Although these RNAs may be expressed at levels that are orders of magnitude lower than certain miRNAs and other highly expressed RNAs, our data reveals the existence of multiple layers of cellular small RNAs, which are likely to be specifically processed and maintained in the cell.

## Results

### Library preparation

To address fundamental questions about the existence and variety of small RNAs in mammalians, as well as miRNA, we prepared two small RNA libraries. We used HepG2, a cell line derived from human liver carcinoma, because small RNAs from cultured cells can be obtained with a minimal risk of contamination due to non-specific degradation of longer RNAs. Small libraries were prepared from RNA extracted from HepG2 cell lines and by ligating the RNA-oligonucleotide adapters to the total RNA, followed by a size based separation (See Methods and additional file 1 for details). Using this cloning strategy, we focus on molecules that have a phosphate at the 5' end and a OH group at the 3' ends, like the known miRNAs. These would not include the non-phosphorylated degradation products. Such RNA molecules, which might have different groups at the 5' and 3' ends, will not be identified with this strategy and will be subject of future studies.

We decided to analyze not only the miRNA fraction (20–25 nt), but also a fraction of slightly longer RNAs (up to 40 nt). The former is expected to represent miRNAs mainly due to i) the size constraint and ii) our protocol which clones 5'-end phosphorylated RNAs. The latter fraction has not yet been characterized by large-scale sequencing and may contain new classes of RNAs, which have not been explored in depth so far. To further explore these potentially novel small RNAs, we prepared two libraries: i) the fraction likely containing miRNAs (19–25 nt) and ii) a broad 30–40 nt fraction, referred here as "short" and "long" libraries, respectively.

Small RNAs may be relevant even if their expression levels are lower than the highly expressed miRNAs, as large mRNAs can often function successfully even with low copy numbers. We concatenated the obtained small cDNAs, cloned them into plasmids and subjected the libraries to the RIKEN RISA large-scale sequencing pipeline, based on Sanger sequencing [18]. We obtained 20,712 and 15,342 sequences from the short (19–25 nt) and long (30–39 nt) RNA fractions, respectively.

### Sequence classification and library quality

**Figure 1:**
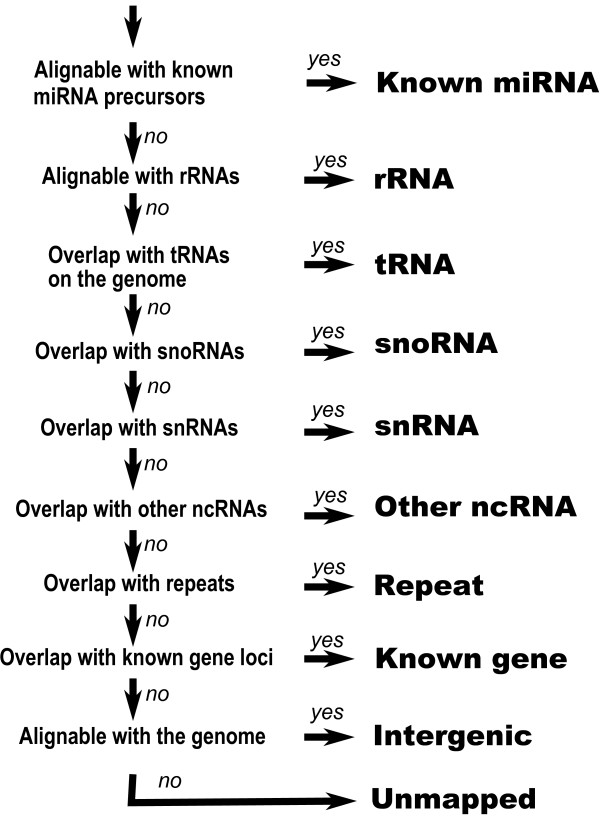
**Classification flow of small RNAs.** Some sequences are alignable with several classes of RNAs and multiple loci on the genome. We assign one sequence to one class based on this flow.

**Figure 2:**
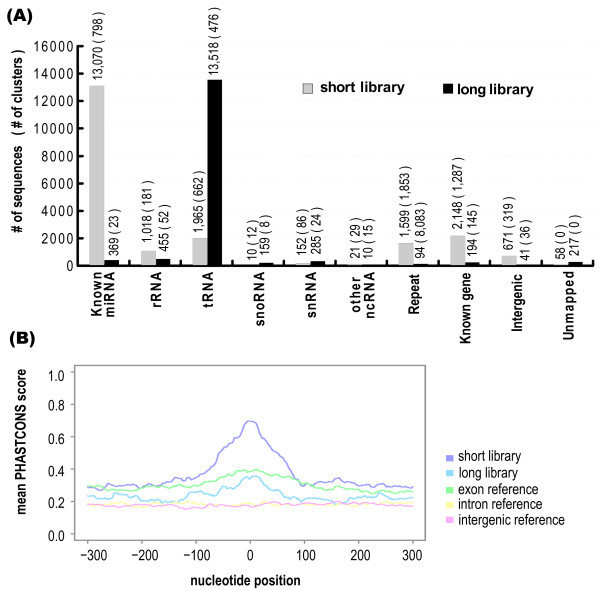
**Content and conservation properties of each sequenced fraction**. a) The count of small RNAs mapping to specific types of annotation is shown as histograms for the short and long libraries. Classifications are based on the process described in Fig 2 and Methods. b) The mean PhastCons [21] scores for the region -300 to +300 relative to the midpoint of each small RNA cluster are shown, broken up into two fractions. Position 0 indicates the midpoint of each cluster. For comparison, 500 equally sized randomly selected regions where the midpoint was either exonic, intronic, or intergenic was analyzed in the same way. The small fraction is generally highly conserved, which is expected due to many potential miRNA sequences it contains. The longer fraction is on average less conserved than exonic sequences, but considerably higher conserved than intergenic or intronic regions.

We classified the sequenced RNAs based on sequence comparison and genome annotations. In the alignment of the RNA and the genome sequence, we used exonerate [19], which approximates a dynamic programming algorithm, and discarded poor alignments which cover less than 85% of the RNA or which sequence identities are less than 90%. Subsequently, we picked up only the best alignments for the classification. This criterion was adopted due to potential biological variation, RNA modification, and errors during the experimental protocols (See Fig 1 and Methods for details). Besides miRNAs, we found considerable amounts of other RNAs derived from transfer RNA (tRNA), ribosomal RNA (rRNA), small nucleolar RNA (snoRNA), small nuclear RNA (snRNA), known gene loci, and transposable elements. The number of RNAs belonging to each class is shown in Fig 2, demonstrating striking differences between the short and long libraries (See Methods for classification criteria).

If random degradation or contamination during library preparation are enriched in our libraries, rather than 5'-phosporylated small RNAs constituting cellular RNA, we would expect higher ratio of rRNAs due to the high constitution (> 80%) of the total cellular RNA. However, this is not likely to be the case; the constitution of rRNA fragments in our libraries is just a minority of the small RNA content. The sequenced RNAs corresponding to the rRNAs constitutes only 5% (1,018/20,712) of the short library and 3% (455/15,342) of the long library, which is considerably lower than the hypothetical distribution deriving from random RNA cleavage. In a previous study of miRNA in a human cell line, HeLa, the fraction of known ncRNAs including rRNA, snRNA, and tRNA was 24%, while the ratio in our short fraction library in our study (which corresponds to the equivalent fraction) was 15% (5% for rRNA; 10% for tRNA; the few other ncRNAs do not contribute significantly) [3]. This suggests that the our libraries include few artifacts resulting from early stages of library construction.

### Evolutionary conservation

The general evolutionary conservation level of functional ncRNAs varies considerably among RNA classes [20]. Therefore, lack of conservation does not necessarily mean lack of function; nonetheless, observed conservation implies selective pressure and thereby function. For the long and short libraries, we investigated the evolutionary conservation level of each small RNA cluster and surrounding genome sequence using mean PhastCons scores [21] (based on genome-wide alignments of sequenced vertebrate genomes) (See Fig 2 and Methods). The small fraction is highly conserved, while the longer fraction is less conserved than typical exons but more than intergenic or intronic sequences. The high conservation of the RNA sequences from the small library is consistent with the high miRNA content in the library; as noted in [20], miRNAs are generally more conserved than other ncRNAs. A large part of the long library consists of tRNA-derived sequences (Fig 2). Such sequences are often labeled as repeats, and thus ignored by PhastCons. Nevertheless, the moderate increase of conservation at the long fraction RNA loci suggests that non-tRNA sequences are under higher selective pressure than their proximal regions.

### Sequence length preferences

**Figure 3:**
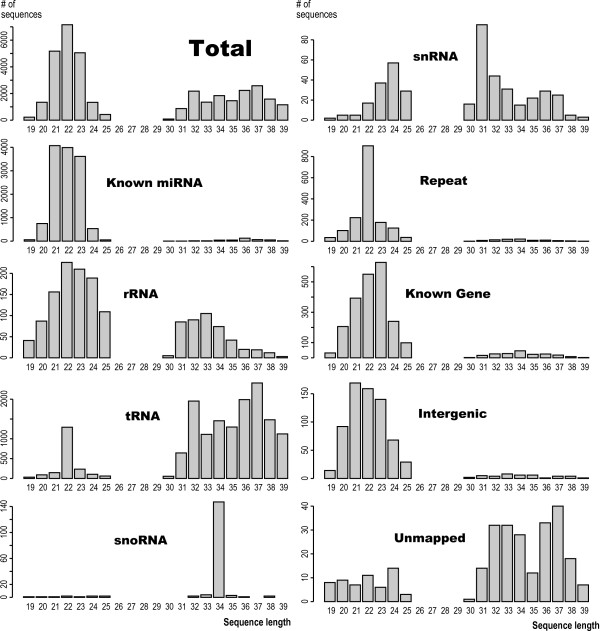
**Length distributions of small RNAs based on our classifications.** Note that the bimodal length distributions of the total small RNAs can be due to our library construction protocol, where shorter and longer fractions are prepared separately. The length distribution per each RNA class demonstrates distinct preferences between these classes.

The short fraction contains sequences with a median length of 22 bp, while the large fraction predominantly contains sequences with lengths of 32 and 37 bp (Fig 3). Each of the small RNA classes has a distinct length distribution (Fig. 3), which argues against random cleavage of RNA precursors. Together with miRNAs (21–23 nt), two additional classes, tRNAs and transposable elements, show peaks centered around 22 nt. Although some repeat-derived small RNAs have been reported as rasiRNA (repeat-associated siRNA) from plants to mammals [22-29], identifying tRNA products of this length was unexpected.

Small RNAs assigned to snoRNAs and snRNAs show peaks at 34 nt and 31 nt, respectively, whereas their full-length cognate forms are longer (from 60 nt to 300 nt). There is also a difference between the lengths of small RNAs mapping to known genes and those mapping to intergenic regions. The former group shows a peak at 23 nt, while the latter shows a peak at 21 nt. Although these classes of RNAs are not as abundant as tRNA-derived small RNA, the length bias depending on RNA species suggest independent mechanisms of controlled processing by specific enzyme producing 5'-end phosphorylated RNAs, such as RNase III, or specific stabilization of randomly processed RNA followed by and/or coupled with some uncharacterized 5'-end phosphorylation mechanisms.

### Uncharacterized pathways associated with tRNA processing

A substantial number of small RNAs are assigned to the tRNA category, while our small RNAs are shorter than 40 nt and mature forms of tRNAs are between 70 nt to 90 nt long. The identified small RNAs are aligned only to well-defined regions of mature tRNAs. Based on the alignments between tRNAs and sequenced small RNAs, we identify a unique characteristic suggesting independent pathways of tRNA processing. These partial tRNAs are unlikely to be the result of non-specific degradation for two reasons: i) lack of correlation to tRNA expression and amino acid usage and ii) specific cleavage patterns. We describe our analysis of these properties below.

#### Expression correlation

If the small RNAs matching tRNAs are the result of a non-specific degradation process, the expression levels of such RNAs should correlate to that of the corresponding tRNA genes. Dittmar et al [30] have presented an effort to profile relative tRNA expression in different tissues using microarrays. Unfortunately, we cannot compare our results directly to this data since our expression data is not relative – it is an estimation of the abundance based on the small RNA counts in one library. An earlier work suggested that the tRNA abundance correlates with the number of tRNA genes in *Caenorhabditis elegans*, as a result of evolution to optimize the efficiency of translation, based on an evidence that tRNA gene number correlate with amino acid frequency in *C. elegans *[31]. Accordingly, we have compared the frequencies of small RNA sequences mapping to tRNAs per isoacceptor family (a group of tRNA genes associated with the same amino acid) with tRNA gene copy numbers (deriving from tRNA gene copies from GtRNA database [32,33] and amino acid frequencies (deriving from Codon Usage Database [34]) (Table 1). The small RNA frequencies have no correlation with tRNA gene copy number or amino acid frequency distribution (Pearson correlation coefficient values are -0.028 (*P *= 0.91) and -0.85 (*P *= 0.72) respectively, where *P*-values are computed based on a null hypothesis that compared distributions have no correlation), while tRNA gene copies correlate with amino acids with Pearson correlation 0.67 (*P *= 0.001). This implies that the cleavage is directed towards certain tRNA types, rather than being a function of tRNA expression. For instance, our libraries identified 4,080 His tRNA cleavage products (on average, 370.9 small RNA reads per each tRNA gene), while the number of Ser tRNA cleavage products was only 17 (0.6 small RNA reads per gene). Although the tRNA copy number is an indirect way of measuring tRNA expression and thus should be treated with caution, the discrepancy in proportions is also demonstrated in the Northern blotting experiment (see below), and this suggests that tRNAs are differentially cleaved and/or their products have differential stability in the cells.

**Table 1:** Small RNAs mapped on tRNAs

tRNA	Major location	Major library	# of small RNA sequences	Amino acid frequency (ratio)	tRNA copies
Lys	5'-end	Long	4183	2176460 (0.056)	34
His*	5'-/3'-ends	Long	4080	1003346 (0.026)	11
Ile*	3'-end	long	1285	1712123 (0.044)	24
Glu*	3'-end	short	1005	2648589 (0.068)	25
Ala	3'-end	long	974	2696299 (0.070)	43
Asp*	3'-end	short	697	1815881 (0.047)	18
Thr	5'-/3'-end	long	687	2053702 (0.053)	24
Arg	5'-/3'-ends	long	576	2193876(0.057)	29
Asn	3'-end	long	477	1392573 (0.036)	29
Gln	5'-/3'-ends	long	426	1797262 (0.046)	32
Pro	center, 3'-end	long	377	2366359 (0.061)	21
Gly	center, 3'-end	short/long	230	2552265 (0.066)	31
Met	5'-/3'-ends	long	167	853648 (0.022)	20
Cys	3'-end	long	161	894927 (0.023)	30
Tyr	3'-end	long	152	1062246 (0.027)	15
Leu	5'-end	short/long	102	3877199 (0.100)	38
Val	-	-	50	2353706 (0.061)	32
Trp	-	-	23	510256 (0.013)	9
Ser	-	-	17	3136765 (0.081)	28
Phe	-	-	8	1465755 (0.038)	12

*... Small RNA sequences subjected to Northern blot

#### Specific cleavage patterns

**Figure 4:**
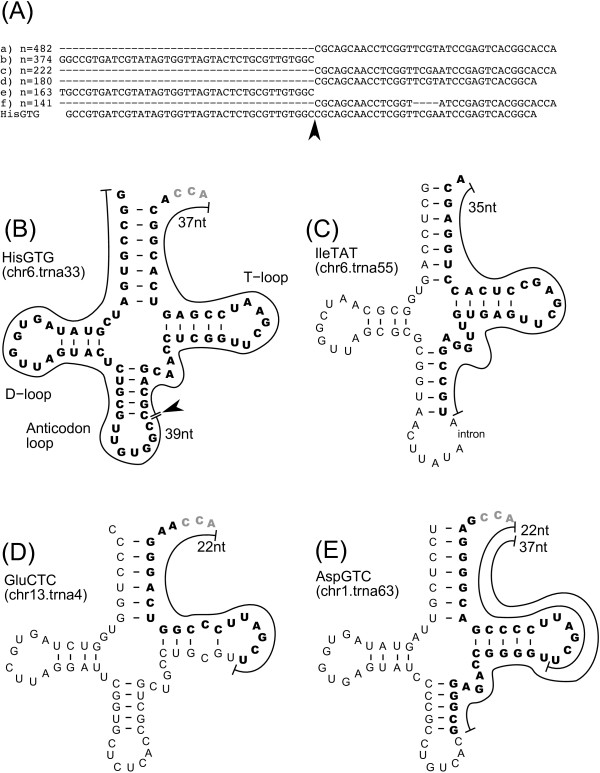
**Small RNAs assigned to tRNA.** (A) An alignment of small RNAs assigned to HisGTG tRNA. Only the top six sequences are aligned with the complete sequence of His tRNA. (B) – (E) Schematic representation of tRNA cloverleaf structures, which are obtained from the Genomic tRNA database [32], and aligned regions of major small RNAs assigned to the tRNA. Bold font indicates small RNA sequences. Gray color indicates added "CCA" in tRNA maturation process, which is not encoded in the genome.

Intriguingly, we found a cleavage at specific tRNA sites: the anticodon-loop and T-loop region within the tRNA cloverleaf structure. An example of the former case is the His tRNA cleavage pattern. RNA molecules of 37 nt and 39 nt long are observed from 3'- and 5'-end of the tRNA. The end of these two products are adjacent to each other at a specific site at the anticodon-loop (Fig 4(A, B)). This exact cleavage and maintenance of its products is strongly supported by hundreds of sequence reads from the long fraction. Precise cleavage at the same site occurs also in Ile tRNA. However, this is different from His as only 3'-end products of Ile tRNA were found in our libraries and its 5'-ends were not found (Fig 4(B, C)). Another cleavage at the T-loop region produces RNAs that are exactly 22 nt long. This cleavage is found in Glu and Asp tRNA, and only their 3'-ends are identified in our libraries. The Asp tRNA has a unique cleavage pattern at the anticodon-loop (Fig 4(D, E)). The length distribution patterns of tRNA derived small RNAs show distinct tendencies between isoacceptors (additional file 2), demonstrating distinct specific cleavages depending on tRNA species.

**Figure 5:**
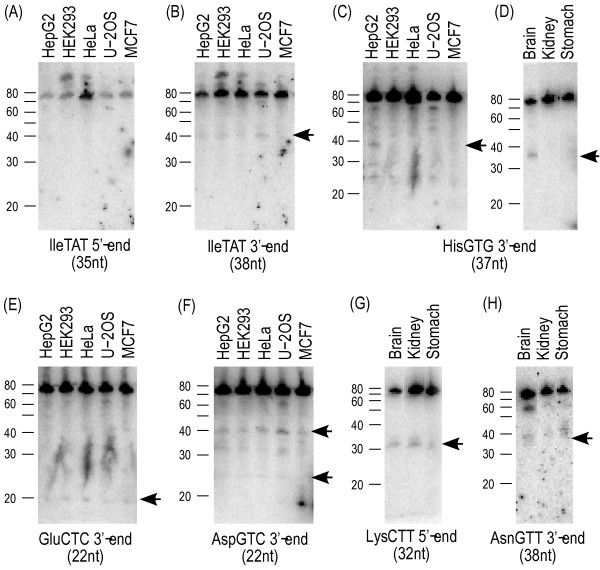
**Northern blotting of tRNA fragments for some tissues and cell lines.** Arrows show the bands corresponding to the small RNA sequences assigned to tRNAs. See the Methods section for the detail of primer sequence and RNA samples.

To confirm these cleavage products by independent means, we performed northern blotting (Fig 5). The results are consistent with our findings using the sequenced small RNAs: the 3'-end products of His and Ile tRNA were detected as 40 nt RNAs, while the 5'-end of Ile tRNA was not found. This demonstrates that the cleavage of tRNA occurs in living cells. The northern blot could not detect the small RNAs corresponding to the Ile tRNA 5'-end, which suggests that only the corresponding 3'-end RNA is maintained in the cells. These findings are consistent with the sequencing of our libraries, and Fig 5 also shows tissue specificity of the tRNA products. Intriguingly, the His tRNA products are clearly detected in HepG2 and brain, but their presence is uncertain in other tissues and cell lines tested. The LysCTT 5'-end is clearly detected in brain, kidney, and stomach, while the abundances of the mature form of Lys tRNAs are not drastically different from the HisGTG. The cleavage events producing 22 nt RNAs are also supported by northern blotting (Fig 5(E, F)).

The observed cleavage events can be interpreted in several ways: (1) controlled cleavage of tRNA and/or specific maintenance of the cleaved products, (2) random degradation of tRNAs at the exposed anticodon loop, (3) artifacts during RNA cloning caused by inefficient reverse transcription at the modified nucleotides, (4) artifacts due to the limitation of our libraries, which cover only 20 nt–39 nt and cannot cover longer products of tRNA. In case (2) (random degradation), we would expect that the abundance of tRNA products correlate with the abundance of tRNA, which is not the case, as described above. The northern blotting confirms this; the abundances of HisGTG 3'-end and LysCTT 5'-end products do not correspond to the full-length tRNAs in brain and kidney (Fig 5(D, G).). The case as in (3) is unlikely, because ligation of adaptors to the RNA is performed before the reverse transcriptase step. RNA molecules causing stops of the reverse transcription cannot appear in our libraries, since RNA sequences considered in this study carry the processed linker sequences ligated at both ends, whereas our reverse-transcription can incorporate mismatched nucleotides at very high efficiency [35]. The northern blotting also rejects the possibility of the case (4), as the cleavage products are detected in both the sequencing and the northern blotting. The Ile tRNA 5'-end, which is 40 nt long RNA potentially and might not be included in our libraries, is not detected even by the northern blotting (Fig 5(A)). The correlation between our sequencing and northern blotting strongly suggests that the small RNA corresponding to the Ile tRNA 5'-end is not maintained in the cell. Therefore, we concluded that our first hypothesis is the most likely: the tRNA cleavage processes are controlled by the cell, and/or its products are maintained in a tissue-specific manner. Notably, we have found no specific base-pattern sites for the cleavage in anticodon loop, but the cleavage separates the conserved U-U dincucleotide when it takes place in the T-loop. It suggests either that different enzymes with different sequence specificity are responsible, or that the exact cleavage is determined based on the RNA structure of the tRNA loop and not the actual sequences. These pieces of evidence suggest the existence of unknown pathways of tRNA processing, where the cleavage patterns are distinguishable between tRNA species.

### 22 nt RNAs derived from LTR antisense or tRNA

About 8% (1,599/20,712) of the RNAs from the short library and 0.6% (94/15,342) from the long library are derived from transposable elements and have distinct length distributions depending on families (additional file 3). The most abundant class within transposable elements is the LTR (Long Terminal Repeat transposons), but not LINE (Long Interspersed repeat Element) or SINE (Short Interspersed repeat Elements), despite the fact that SINEs are the most abundant repeats in the genome. Furthermore, the small RNAs are in opposite (antisense) orientation to LTRs and LINEs, whereas Alu derived RNAs appear in the same orientation to the repeat element sequence. Finally, the length of small RNAs aligned with LTRs and LINEs is exactly 22 nt, resembling the size of miRNA, siRNA, and a subset of tRNA cleavage products.

Each of the three LTR families consists of a remarkably abundant (representative) sequence and considerably fewer minor variations including 5'- and 3'-end variations, which might be caused by imprecise processing and/or by expression of repeats diverging from the consensus sequence. Intriguingly, the representative sequences derive from the antisense strand of LTR, and they are very similar to tRNA 3'-end. Alignments of the representative small RNAs, complementary sequences of LTRs, and tRNA 3'-ends (additional file 4) show that the tRNA sequences differ from the other two sequences just in two positions. The first is a mismatch from "A" to "U" at the 57th or 58th adenine on the tRNA, T-loop. The second difference from the tRNA is that the putative repeat-derived small RNA has the trinucleotide signature "CCA" at the 3'-end. 1-methyladenosine at position 58 (m^1^A58) of tRNA is reported in many organisms including human [36,37] and therefore the m^1^A58 might have been copied into "U" during the reverse-transcription of our library preparation in 499 cases, versus 453 sequences where it reads as an "A". However, a such high frequency of purine-purine misincorporation during reverse transcriptase seems improbably high if compared to experimental evidence [38], and the fact that in the tRNAs the m^1^A58 still pairs with an "U" [39]. The "CCA" is added during the maturation process of tRNAs [40]. Therefore, two hypotheses can be made about their origins: they could be i) antisense transcripts from LTRs or ii) tRNA cleavage products whose sequence can be aligned with LTR antisense, which could be derived from multiple polymorphic loci. The existence of siRNA derived from transposable elements was reported as repeat-associated small interference RNA (rasiRNA) in various organisms [22-25] and was only recently started to be identified in mammals [26,27]. These originate from various scattered regions within repeats rather than from a specific site, and are generally represented on both strands. In contrast, the small RNAs described above are putatively derived from just the antisense strand of specific region in the transposable elements. Therefore, besides being rasiRNAs, these RNAs could be the unprocessed variants of the tRNA cleavage products.

### C/D box type snoRNA and snRNA products

Small nucleolar RNAs (snoRNA) are between 60~300 nt long RNAs, and contribute mainly to 2'-O-methylation and the pseudouridylation of rRNA, snRNA, and potentially other RNAs. Some small RNAs, 0.05% (10/20,712) from the short library and 1% (159/15,342) from the long library are cleavage products of the corresponding mature snoRNAs. Surprisingly, 90% of these snoRNA cleavage products (152/169) are derived from a single gene: the C/D box type snoRNA gene mgU6-77 [41,42] (additional file 5). The small RNAs match 34 nt sequence just at their 3'-end.

Small nuclear RNAs (snRNAs) constitute a class of small RNA (100 to 300 nt) localized in the nucleus, where they form small nuclear ribonucleoprotein (snRNP) that are involved in RNA splicing. Some small RNAs, 0.7% (152/20,712) from the short library and 1.4% (285/15,342) from the long library, correspond to snRNA. Their length distributions show peaks at 24 nt and 31 nt in the short and long library, respectively (Fig 3). All of these small RNAs correspond to specific 3'-end region of U1, U4 and U5 (additional file 6), while no snRNA products derive from the other regions. These products of longer well-known RNAs, snoRNA and snRNA, also suggest a controlled cleavage or stabilization of its products, which is analogous to but distinct from the suggested pathways of tRNA cleavage.

### Small RNAs overlapping known genes

A substantial number of small RNAs derive from known gene loci. There is a remarkable difference in sequence lengths between RNAs derived from sense and antisense strands (additional file 7). The most abundant lengths of RNAs derived from introns are 21 nt and 23 nt for the sense and the antisense strand, respectively. Their length distributions are significantly different (*P *= 4.3e-10, two-sided Mann-Whitney test). There is also a remarkable difference between 3'UTR and introns in terms of small RNAs mapping to the antisense strand. The most frequently occurring lengths of such RNAs are 24 nt and 23 nt, respectively (*P *= 2.2e-16, two sided Mann-Whitney test).

**Figure 6:**
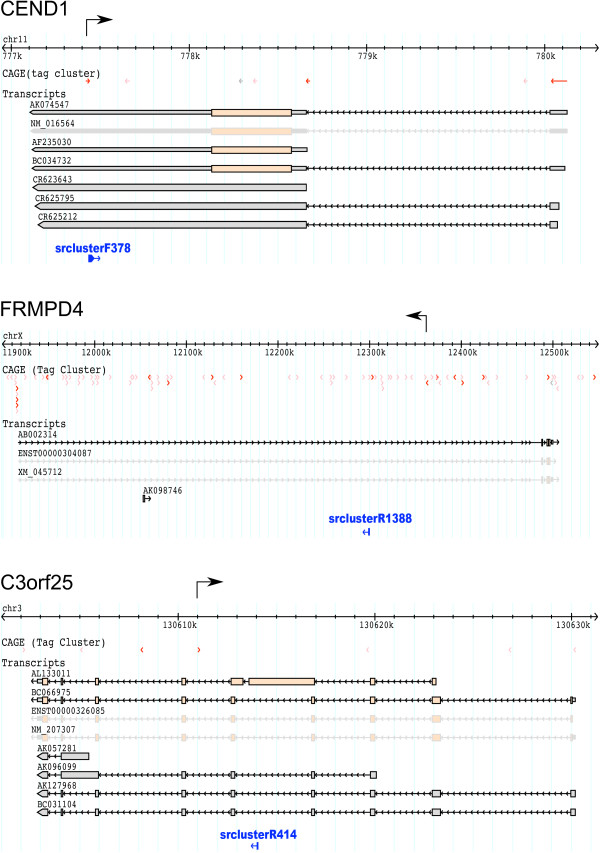
**Schematic representation of genomic regions, where the small RNAs assigned to known genes are mapped on.** Clusters of the small RNAs are represented in blue, transcription starts captured by CAGE are represented in pink (supported with single CAGE tag) and red (supported by two or more tags) [45]. Antisense transcription to the gene, which is near to the small RNAs, is represented as arrow on the genome representation.

A possible pathway to produce these small RNAs is the cleavage of long dsRNA that are derived from simultaneous transcription of a region on both strands, which resembles the L1-derived rasiRNAs in human [27]. Interestingly, a substantial number of small RNAs in each subclass are derived from one locus (Table 2): 636 of 1,163 (35%) of all small RNAs from that locus are both intronic and antisense are mapped on the *FRMPD4 *locus (chrX:12299549..12299573), 183 of 664 (30%) of all small RNAs from the intron sense strand are mapped on *C3orf25 *(chr3:130614020..130614043), and 68 of 84 (80%) of all small RNAs derived from the 3' UTR antisense strand are mapped on *CEND1 *(chr11:777438..777463). To examine the hypothesis that those small RNAs are processed products of sense-antisense derived dsRNAs, we used CAGE (Cap-analysis of Gene Expression) tags, which are 5'-end reads of full-length cDNAs by capturing 5'-capped sites [43,44]. In a previous study, more than 5 million CAGE tags were sequenced and mapped onto the human genome sequence [45,46]. Using the CAGE data, we found evidence of sense-antisense transcription on both strands for these three loci (Fig 6). These findings are consistent with the above hypothesis of dsRNA originated small RNAs (See also below for the related section on bidirectional promoters).

**Table 2:** Known gene derived small RNAs

**subclass of known gene derived small RNA**				**The most abundant clusters**				
**Relative Location to the gene**	**Orientation**	**# of RNA**	**# of clusters**	**ID**	**sequences**	**Locatin**	**gene**	**major length**

Intron	sens	664	340	srclusterR414	183 (28%)	chr3:130614020..130614043	C3orf25	21 nt
Intron	antisense	1163	441	srclusterR1388	636 (55%)	chrX:12299549..12299573	FRMPD4	23 nt,22 nt
3'UTR	antisense	84	22	srclusterF378	68 (81%)	chr11:777438..777463	CEND1	24 nt

We cannot rule out other explanations at this moment. One of them would be cleavage of folded RNAs harboring dsRNA. To examine this possibility, we checked local secondary structures of these loci and found that the potential precursor of the small RNAs located in CEND1 locus can comprise a secondary structure harboring the dsRNAs, where cleavage of the dsRNA could produce the small RNAs (additional file 8).

### Bidirectional promoters

One of the sense-antisense relationships that have received considerable interest in recent years is the bidirectional promoter, where two closely located promoters are on opposite strands. These have been found to be abundant in mammalian genomes [47]. CAGE data indicating transcription starting sites (TSS) in this orientation occur more often than previously appreciated [45,48].

**Figure 7:**
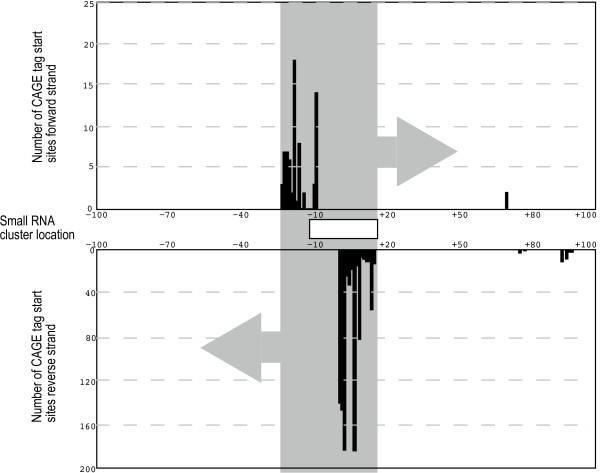
**Example of small RNAs overlapping a bidirectional promoter.** The histograms show frequencies of transcription start sites (TSS) captured by CAGE. Top and bottom panels show CAGE tag-defined TSS mapping to forward and reverse strand, respectively. The shaded grey box indicates the region with potential for forming a dsRNA by transcription on both strands. The location of the small RNA cluster inside this region is indicated in the central panel as a white box. The numbering of genomic nucleotides is assigned by defining the center of the small RNA cluster as +1.

For clarity, bidirectional promoters can produce transcripts that can be overlapping – this occurs when the promoter on the reverse strand is located downstream with reference to the other promoter. A large subset of bidirectional promoters have this property, producing transcripts that are overlapping but on different strands, and giving rise to potential formation of dsRNA by sense-antisense hybridization, which might be a regulatory event in itself. We reasoned that some of the small RNAs identified in this study could be derived from such dsRNA formation, analogous to the *FRMPD4 *locus case above. Using CAGE, we identified overlapping bidirectional promoters where there are at least two TSSs on opposite strands within 40 bp, and where the two transcripts can potentially hybridize. Such bidirectional promoters could principally be located further away than 40 bp: however, we choose 40 nt cutoff since CAGE tags have a characteristic length of 20–21 bp and thus the tags under analysis are guaranteed to cover the entire region between the TSS on both strands. This makes overlapping transcripts likely. Since we focused our study on strong promoters, we only assessed TSS having at least 10 CAGE tags. Using these criteria, we obtained 93 overlapping proximal bidirectional promoters. There are 12 clusters of our small RNAs that directly mapped on these 93 bidirectional promoters (additional file 9). In most cases, the small RNA clusters are located between the TSSs (Fig 7), consistent with the dsRNA hypothesis. This outcome is highly unlikely to happen by chance (*P *< 10E-3) judged by randomization trials (See Methods): although correlation does not necessarily imply causation. However, if the causation is true, this would mean that the bidirectional promoters can function as an origin of natural dsRNAs that are specifically cleaved.

Apart from the significant overlap with the small RNAs, we noticed a second interesting feature associated with these bidirectional promoters: a partial but exact match of both CAGE tags (detected on both RNA strands) and small RNAs to ribosomal RNA sequences (only the strand corresponding to ribosomal RNA detected, not the antisense; additional file 10). There are several clusters of rRNA genes (or rDNA) tandemly repeated hundreds of times [49], while ribosomal RNA sequences are not part of the current genome assembly (hg17). Partial copies of rDNA occur in the genome assembly, and the small RNAs within bidirectional promoters are located in these interstitial rRNAs. Due to the (partial) sequence identity between the interstitial rRNAs and rDNA repetitive clusters, we cannot unambiguously conclude about the origin of our small RNAs and CAGE tags. It is possible that our small RNAs and CAGE tags are degradation products caused by abundant ribosomal RNA within a cell. However, the CAGE tags on the antisense strand are unlikely to be such degradation products, because the CAGE method does strictly maintain the strand directionality, as demonstrated by its capability to detect sense-antisense RNA transcription [50]. Note that typical rRNA should not be captured by CAGE because that rRNA is transcribed by polymerase I, not II, and have no cap structure at its 5'-end. These evidences underline the complexity of the transcription and/or processing pathways of rRNA or rRNA-related sequences.

To examine whether these are general characteristics to the interstitial rRNA, we identified 140 regions on the genome assembly, which are similar to or partially identical to rRNA as identified by using BLAT [51] (additional file 11). Of the 140 interstitial rRNA, 55 (39%) and 64 (46%) express small RNAs of the same strand to the rRNA and bidirectional transcription (CAGE tags are mapped on sense and antisense strand of the interstitial RNA) respectively, and 37 (26%) encode both of them. It is possible that the two characteristics, small RNA encoding and bidirectional transcription, are a generic feature of interstitial rRNAs. If we assume that the small RNAs are derived from rDNA repetitive clusters, then they can be considered as rasiRNA. Selective regulation of histone lysine methylation was reported on mouse rDNA [52], and these small RNAs might be involved in epigenetic modification.

### Novel miRNA candidates

Over 60% of the sequences in the short library can be assigned to known miRNAs (Fig. 2). This is consistent with previous findings [4,53], which demonstrates that our experimental procedure has succeeded to identify endogenous small RNA in human cells. In order to discover novel miRNAs, we applied standard criteria for miRNA annotation [54], consisting of expression and biogenesis. Since the sequences in our libraries are expressed (as we detect them by sequencing), we focused on predicting possible folding patterns. We did not use conservation as a criterion for identifying miRNAs, because there are specie-specific RNAs that are not significantly conserved even in the recent primate evolution [53]. There are 3,336 novel sequences that did not correspond to any known miRNA, tRNA, rRNA, transposable elements, or other ncRNA, which are located in 1,557 clusters of small RNAs. We inspected genomic regions proximal to these clusters and determined whether they can fold as hairpin-loop precursors, whether their stem regions correspond to the sequenced RNA, and whether their structures are conserved among several species. Finally, we found six novel miRNA loci potentially encoding miRNA precursors, from which 20 nt–21 nt mature miRNAs corresponding to the small RNAs in our library can be cleaved out (see Table 3 and additional file 12; additional file 13 for predicted secondary structures).

**Table 3:** Novel miRNAs

**miRNA name (cluster name)**	**mature sequence of novel miRNA**	**reads**	**Predicted precursor of novel miRNA location / comments**
hsa-mir-1469 (srclusterF2762)	CUCGGCGCGGGGCGCGGGCUCC	1	chr15:94,677,494-94,677,540 (+) / intron of NR2F2
hsa-mir-1251 (srclusterF3130)	ACUCUAGCUGCCAAAGGCGCUU	1	chr12:96,388,159-96,388,215 (+) / intron of RMST
hsa-mir-1470 (srclusterF891)	GCCCUCCGCCCGUGCACCCCG	1	chr19:15,421,359-15,421,419 (+) / antisense intron of WIZ
hsa-mir-675b (srclusterR1978)	CUGUAUGCCCUCACCGCUCAG	3	chr11:1974572-1974628 (-) / star sequence of miR-675
hsa-mir-1471 (srclusterR2035)	GCCCGCGUGUGGAGCCAGGUGU	1	chr2:232,582,457-232,582,513 (-) / intergenic
hsa-mir-1468 (srclusterR3487)	CUCCGUUUGCCUGUUUCGCUG	1	chrX:62,788,915-62,788,976 (-) / intergenic

Intriguingly, a novel miRNA and its complementary counterpart within the precursor stem are derived from the first exon of an imprinted non-coding gene H19, which is expressed from the maternal allele during development [55] (additional file 14). During the preparation of this manuscript, the counterpart was reported as miR-675 in the 293T cell line [56]. The miRNA is sequenced only once from our library, and the novel miRNA ("star" sequence of the miR-675), miR-675b, is sequenced three times. These findings indicate that the H19 derived miRNAs varies depending on its context or on the tissue it is expressed in.

## Discussion

We have investigated the existence of small functional RNAs in the HepG2 cell, a liver carcinoma cell line. By taking precautions to minimize artifactual random RNA degradation, our data demonstrates that besides new miRNAs, there are further classes of small RNAs that have not yet been properly described. Previous studies analyzed small RNAs similar in length to the short fraction (19–25 nt) [4-6,8]. They mainly focused on RNA-silencing related RNAs, miRNA and on siRNA, and did not further analyze other RNAs after classification. No studies have examined small RNAs equivalent to the long fraction (30–39 nt), except for piRNA [12-15].

The first surprise is the content of the short and long fractions, revealed by classification of the sequenced small RNAs. The main content of the short fraction is miRNA, and this observation is consistent with previous studies. Unexpectedly, the long fraction consists mainly of tRNA. The classification also shows that a substantial number of RNAs is derived from intergenic regions. This is consistent with previous reports showing that at least 60 – 70% of the genome is transcribed [46,57].

### Potential tRNA cleavage pathways

A surprising class of small RNAs is composed by short forms of tRNAs. Detail examination of the sequences and the subsequent experiments suggest that the sequenced RNAs are likely to be non-random cleavage products of tRNA. This leads two hypotheses which are not mutually exclusive: (1) the cleavage products are functional by themselves and produced intentionally, (2) the products are the result of a controlled cellular process, which has not been reported previously. We did not examine these hypotheses here with targeted experiments; they remain to be answered in future works.

The cleaved products do not resemble a result of tRNA degradation by the nuclear surveillance pathway shown in yeast, where defective tRNAs are at first polyadenylated, followed by exosome processing [58,59]. Some RNases are known to cleave tRNA internally, such as Onconase [60] (an RNase found in eggs and early embryos of the leopard frog that cleaves tRNA specifically at UG and CG, and GG dinucleotides) and M1RNA [61,62] (RNaseP in *E. coli*), but there are no reports of such endogenous RNase activity in human. Another possible enzyme to cleave out the small RNAs (especially for the product with the same size to the siRNA and miRNA) is an RNase III enzyme, such as Dicer, which cleaves dsRNAs and produce 5'-end phosphorylated RNAs. To examine this possibility, we analyzed the nucleotide composition of the tRNA products (additional file 15), since cleaved products of RNase III enzymes tend to have "U" at the 5'-end [17,63]. We found a clear bias towards "U" at the 5'-end in the 22 nt long products. After detailed assessment of the tRNA sequence, the high frequency of "U" at the 5'-end of 22nt RNA arise from a nucleotide corresponding to the position 54 of tRNA, which is commonly "U" in all of the tRNAs. Thus, this bias does not strongly support the hypothesis that the cleavage enzyme is of the RNase III type, although on the other hand it does not conflict with this hypothesis. Another possible pathway is the association with LTR's primer binding sites, although this cannot explain all of the cleavage because the tRNA cleavage occurs in tRNAs which are not used as primers (additional file 16). In particular, the 22 nt sequences resembling tRNA and LTR antisense exactly correspond to 3'-end region of tRNA, which can hybridize LTR primer biding site (additional file 4). The base-pairing of tRNA and LTR could contribute to the 22 nt RNA production from tRNA.

A possible effect of tRNA cleavage is indirect silencing, which is best explained by an example, Onconase. Cancerous cell line growth is suppressed with a delay of 24–48 hours when the cells are treated with Onconase [60]. This delayed response lead to the hypothesis that growth suppression is not caused by direct toxicity due to protein synthesis inhibition caused by tRNA cleavage, but through the production of secondary messages (the cleavage products of tRNAs) that would interfere with yet unknown cellular mechanisms [64]. Similarly, human RNases of yet unknown functions may contribute the specific cleavage of tRNA to produce new RNA effectors. A candidate for such an effecter is the 22 nt cleavage product of tRNA, 3'-end of Glu tRNA, which might be involved in RNA silencing in a similar way to miRNA and siRNA. Based on the sequence complementarity, possible targets for silencing are transposable elements that use the tRNA 3'-ends as primers. Besides the fact that RNA silencing is used in many organisms to protect the host genome from retroviruses and retrotoransposons, the use of tRNA would have additional benefits such as being readily available and being effective: tRNA is abundant within a cell and active transposable elements to be repressed should have a priming site for reverse transcription. Another possible machinery to suppress mRNAs is the use of RNase P and/or RNase Z. mRNAs of influenza virus and HIV-1 can be cleaved by the RNases with external guide sequence (EGS), which is an RNA similar to the tRNA short form and plays its role as a guide to specify its target RNAs [65,66]. These RNases may use the cleaved product of tRNA demonstrated here as a guide to regulate genes. All of the above hypotheses, based on our novel data, await future experimental validation.

### Overlaps between bidirectional promoters and rRNAs

Besides unknown cleavage of tRNA, snoRNA, snRNA, we found a significant overlap between bidirectional promoters and small RNAs, suggesting a connection between naturally formed dsRNA and cleaved small RNAs. We also found that these regions are located in rDNA repetitive clusters or interstitial rRNAs. This is the first report showing that rRNA or rRNA related sequences can be transcribed in a bidirectional way and, encoding small RNAs, despite mature rRNAs are classical functional RNA studied in-depth previously. Although we cannot conclude the origins of these CAGE tags showing bidirectional transcriptions and these small RNAs due to partially identical sequences, small RNAs are likely to be related with these antisense transcription events. Elucidation of the detailed mechanisms requires further studies. More deep sequencing will be necessary to correlate these short RNAs with the whole genome tiling array studies [67], which identify the signal deriving from short RNAs overlapping TSS of mRNAs

## Conclusion

We have discovered multiple classes of small RNAs derived from many different sources, in addition to six novel miRNAs. Specific cleavage of well-characterized non-coding RNA, such as tRNA, snoRNA, and snRNA, is likely to produce the small RNAs observed in our libraries. Bidirectional (or sense-antisense) transcription might also contribute to their biogenesis. Our new dataset and its analysis show that there are distinct patterns of small RNA biogenesis that greatly expand our knowledge, paving the way to downstream studies to elucidate various complex patterns of RNA expression, cleavage, maintenance and mechanisms of actions. In particular, tRNA cleavage and bidirectional transcriptions within rRNA (or related sequences) underscore the complexity of the transcriptome and add yet another layer to the complexity of transcription in mammalian cells.

Our data also emphasize on the inconvenience and constraints of having hard definitions for biological molecules. Indeed, a part of known RNAs (such as tRNAs, rRNAs, snRNAs, and snoRNAs) is present in multiple forms, which may play different biological roles. These data imply that cells use the same small RNA molecules multiple times for a variety of functions. Multiple roles of the same molecule are widely recognized for mRNA as alternative splicing, and an analogous concept should be applied for entire RNA molecules including mRNA and non-coding RNA. In our study, it was possible to unambiguously distinguish the origin of our small RNAs, between tRNA and antisense of transposable elements, and between frequently repeated rDNA sequence and just partial copies. Small RNA derived from these ambiguous regions is putatively linked to genome and transcriptome evolution. The unrevealed pathways implied in this study underline the importance of a less dogmatic approach to study and annotate the transcriptome and its regulation.

## Methods

### Total RNA extraction

HepG2 cells (from an hepatocellular carcinoma from a 15 years old male) were cultured in Minimum essential medium (Eagle) with 2 mM L-glutamine and Earle's BSS adjusted to contain 1.5 g/L sodium bicarbonate, 0.1 mM non-essential amino acids, and 1.0 mM sodium pyruvate, 90%; fetal bovine serum, 10%. Total RNA was extracted by acid guanidinium phenol chloroform (AGPC), expect that all precipitations were done with ethanol, instead of isopropyl alcohol, to ensure the recovery of small RNAs.

### Small RNA library preparation

A 5' phosphorylated 3' RNA/DNA chimeric oligonucleotide (sequence: 5'-phosphate-UUUaaccgcgaattccag-biotin-3', the upper-case indicates the RNA part of the oligonucleotide, lower-case indicates the DNA part oligonucleotide) and a 5' adaptor DNA/RNA chimeric oligonucleotide (sequence: 5'-acggaattcctcactAAA-3', the upper-case indicates the RNA part of the oligonucleotide, lower-case indicates the DNA part oligonucleotide) were simultaneously ligated to 1 micro gram total RNA with T4 RNA Ligase (Takara), as modified from [3]. Purified small RNAs ligated (insert of the target length + linkers) with both linkers were separated from adaptor dimer (36 bp) on a 5% denaturing PAGE gel.

After the chimeric-oligonucleotide adapter ligation, the cDNA synthesis was carried out from the purified small RNAs after the ligase step above, using 3'RT-PCR primer (sequence:5'-gactagctggaattcgcggttaaa-3') with M-MLV reverse transcriptase with RNase-H-minus, point mutant (Promega). The cDNA derived from small RNA were amplified by PCR using adaptor-specific primers: primer 1 (short-RNA3'RT-PCRprimer): 5'-biotin-gcacgctggcctcgtgagaattc-3'; primer 2 (short-RNA5'PCRprimer): 5'-cagccaacggaattcctcactaaa-3'. PCR was performed from 1 μl of template RT product, 5 μ1 of 10× buffer, 4 μl of 2.5 mM dNTPs, 0.5 μl of 100 uM primer 1, 0.5 μl of 100 μM primer 2, 0.5 μl of EX-Taq polymerase (5 U/μl, Takara) in a total volume of 50 μl. After incubating at 94°C for 1 min, 26 PCR cycles were performed for 20 sec at 94°C, 30 sec at 55°C, 1 min at 70°C; followed by 5 min at 70°C.

The PCR products were further purified on a 12% polyacrylamide gel for separating the two main bands used for the libraries (20–25 and 30–40 nt inserts). PCR-amplified, gel-purified small RNA tags were re-amplified, followed by digesting with *Eco*RI (Fermentas). The desired products were purified on a 12% polyacrylamide gel and after elution were concatenated with T4 DNA ligase (New England Biolabs) for 20 minutes at 16. Proteinase K treatment was carried out, followed by purification and ethanol precipitation and redissolved with 2 μl of distilled water.

The concatenated products was then ligated to 50 ng pZErO-2 cloning vector, previously cleaved with *Eco*RI, and the ligation with the T4 ligase (New England Biolabs) was carried out in 5 μl under standard conditions. The reaction was allowed to proceed overnight at 16°C to ligate concatemers to the vector. Subsequently, the reaction mixture was purified and the DNA was precipitated with ethanol. The DNA was finally resuspended in distilled water, followed by electroporation into DH10b ultracompetent cells (Invitrogen) at 2.5 kv/cm, plated on zeocin plates (50 μg/ml), and the obtained plasmid was sequenced with "forward" primer as described [18]. Sequences of the inserted small RNAs were extracted by identifying *Eco*RI ligated doublet linker (24 bp) with allowing 2 bp mismatch, after vector masking with 12 bp match at least. The sequences have been submitted to the DDBJ (Sequence IDs are ADAAA0000001 – ADAAA0005988, and ADAAB0000001 – ADAAB0006146).

### Genome, miRNA, and rRNA mapping

The first step to characterize the sequenced RNA is to find the loci encoding them. We aligned them with several known sequences, the human reference genome sequence (NCBI build 35, hg17; all of the genomic coordinates in this paper are based on hg17), known miRNA database (miRBase Release 8.2), and ribosomal DNA sequences (X03205, J01866, M11167). The sequenced RNA should exactly match to these sequences in the ideal case, but this is not always correct due to several reasons: (1) Biological variation: the sample used here is a cancer cell line, HepG2, and its genome should have many variations from the reference of human genome (2) RNA modification: some ncRNAs are known to have modifications at specific positions to achieve their function. Modified nucleotides potentially cause mismatches during the experimental process such as reverse-transcription, PCR, and sequencing. Especially tRNA, the major component of the long fraction in this study, has additional trinucleotides "CCA" at the 3'-end as well as many internal modifications [36] (3) Errors during the experimental protocols, such as RT-PCR and sequencing. Thus, we aligned the small RNA sequences with allowing a few mismatches, and picked up the best one from the alignments as the locus producing the small RNAs.

Some widely used local alignment tools, such as BLAST [68] and BLAT [51], are not optimal for mapping small RNAs due to their heuristics. We adopt a sequence alignment program, *exonerate*, which allows approximation to exhaustive alignment based on dynamic programming [19]. The program was run with the following options: "–dnawordlen 8 –model affine:local –score 80 –wordjump 1". In order to exclude poor alignments and reduce ambiguity, we filter out alignments where less than 85% of the RNA is aligned, or the sequence identity is less than 90%. We adopt these parameters to align the tRNA products, which is short but frequently include the RNA modification site and trinucleotides signature of tRNA "CCA" at the 3'-end, to the genome properly. A substantial part of the tRNA products with 22 nt length have the trinucleotides (additional file 17), and the requirement of 85% or more aligned region is adopted to cover those cases (19 nt/22 nt = 86%). After this filtering, we keep only the alignments with the highest score and remove the others. Even after this restrictive procedure, single RNA sequences can be mapped on multiple locations due to the genomic repeats contained in the genome such as transposable elements and tRNA. Hence, our procedure allows multiple mapping. The small RNAs are also aligned with miRNA precursor and rRNA, according to Fig 1. The same criterion to the genome mappingis performed against miRNA hairpin sequences in miRBase and rRNAs.

### Clustering

Based on the genomic coordinates, we defined small RNA clusters on the genome, where sequences overlapping on the same strand with at least one base pair are merged. Associations between small RNAs and these clusters are described in additional file 18, and genomic coordinates of these clusters are described in additional file 19 in General Feature Format (GFF).

### Sequence classification

The small RNAs are classified based on the mapping described above and on genome annotations (Fig 1). If an RNA sequence was aligned with the miRNA or rRNA, the sequences are assigned to these classes. Assignment to miRNA is prioritized because it is the most likely species within these sized of RNA, 20–40 nt. rRNA is used as an indicator of contamination during library preparation, and its assignment is prioritized to avoid under estimation of the possibility. If the sequence is neither miRNA nor rRNA, genomic coordinates and genome annotation was used. We retrieve genomic coordinates of tRNA from the GtRNA database [32,33], snoRNA and repeats from the UCSC Genome Browser Database [69], and transcriptional units (TU) from the FANTOM3 data repository [46]. A TU is a cluster of transcripts which exons that overlap each other, and we use it's genomic coordinates as known gene loci. The small RNAs are assigned to the five classes, snoRNA, snRNA, other ncRNA, repeat, and known gene according to the Fig 1. The two classes intergenic and unmapped are adopted for the rest of sequences, in case that it can or cannot be mapped on the genome, respectively. The result of the classifications is described in additional file 18.

This classification is based on the mapping described above, where the best aligned genomic regions are used even if they are not identical matches to the small RNA reads. Additional file 20 shows the both cases of the classifications of all of the mapped small RNA reads and only the reads that is identical to the genome sequences. This demonstrates that the entire tendencies are unaltered: the main constitutions of the short and long libraries are miRNA and tRNA, respectively. The ratio of tRNA is relatively low when only the identical reads to the genome are used, which is caused by the tRNA modification, such as nucleotides in T-loop and/or "CCA" addition to the 3'-end.

### Conservation analysis

As described above, we investigated clusters of mapped small RNA long and short fractions independently. For each such cluster, we located the midpoint of the cluster, and retrieved PhastCons [21] scores from the UCSC database [70] (hg17 assembly), for the -300 to + 300 nt region relative to the midpoint. PhastCons is a Hidden Markov Model-derived conservation index on the nucleotide level based on all complete vertebrate genomes, where PhastCons scores can be viewed as the estimated likelihood that a nucleotide is under purifying selection, based on a phylo-HMM having two states with two different phylogenetic models correspond to 'conserved' and 'nonconserved' [21,71]. Thus, for each small RNA cluster we obtain a vector of 601 conservation values. From the alignment of all clusters from the same fraction to the midpoint, we can assess the general conservation level of the small RNAs and their surroundings, here expressed as the mean PhastCons score. For comparison, we randomly selected 500 genomic regions of the same size in which the midpoint was intergenic, exonic or intronic, judged from GenBank mRNA mappings, and performed the same analysis. Nucleotides with no PhastCons value (often due to repeats) were treated as missing values in the calculation of the mean.

### Northern blot

RNAs of the following cell lines and primary tissues were used for northern blotting: 293 (from embryonic kidney), HeLa (from cervix carcinoma), U-2 OS (from osteosarcoma), MCF-7 (from breast cancer), brain (Biochain#R1234035), kidney, and stomach. This study is approved by RIKEN Yokohama Institute Ethical Committee (Approved numbers H15-41, H16-7, and H17-46).

For northern hybridization analysis of small RNA, 12.5 μg of RNA was separated by 10% polyacrylamide gel electrophoresis with 7 M urea in 0.5 × TBE solution (100 mM Tris pH 8.0, 90 mM of borate, and 1 mM of EDTA pH 8.0) using XCell SureLock Mini-Cell unit (Invitrogen). The electrophoresis was performed with 20 volts for 30 minutes at room temperature. Then the RNA was transferred to positively charged nylon membrane, Hybond N+ (Amersham) by electroblotting under 20 mA (constant voltage) at room temperature for 1 hour, XCell II Blot Module (Invitrogen).

The probes for northern blotting were labeled with [g-32P]ATP by the use of mirVanaTM Probe & Marker Kit (Ambion). The probes were designed as a complemental to a 3'- fragment of transfer RNAs described in additional file 21.

The RNA blotted on the membrane was hybridized with the synthetic oligonucleotide probes at 42°C for 12 to 16 hours in a hybridization oven (NIPPON Genetics). The membranes were then washed 3 times with northern wash solution (6 × SSC and 0.2% SDS) for 5 minutes at room temperature and washed only once with the solution for 15 minutes at 42°C. The hybridized signals were detected by Bio-Image analysis system (Fuji Film).

### Randomization experiment

Our goal was to assess the likelihood of the observed overlap of the small RNA clusters with the 93 bidirectional CAGE promoters. For this, we randomly selected 93 non-overlapping genomic regions with the same size distribution as the observed bidirectional promoters and observed how many small RNA clusters overlapped these regions, and repeated this procedure 1000 times. We observed no overlap in any randomization trial and conclude that the likelihood is < 10E-3.

## Authors' contributions

HK carried out the main part of the computational analysis and drafted the manuscript. MN carried out the library preparation of the small RNAs. YT carried out the northern blotting; AS carried out the computational analysis of genome conservation and bidirectional promoters. SK and SF assisted in the computational analysis. HK, AS, CD, JY, and PC finalized to the manuscript. CK and JK assisted in the coordination of this study. JY designed and coordinated the northern blotting. PC conceived the study and carried out its design. YH conceived the study. All authors read and approved the final manuscript.

## Supplementary Material

Additional file 1 Schematic representation of the experimental protocols of small RNA library preparation. Ten steps in the protocols are shown. Click here for file

Additional file 2 Length distributions of tRNA derived smallRNAs per isoacceptor. The distributions are shown as histograms, where X and Y axes mean the length of the small RNAs and their frequencies, respectively. Click here for file

Additional file 3 Length distributions of repeat derived small RNAs per repeat classes and strands. Histograms are shown in the same way to the additional file 2. Click here for file

Additional file 4 Relationship between tRNA and LTR. Schematic structure of tRNA priming of LTR reverse transcription (A), and alignment of LTR's primer binding site (PBS), tRNA 3'-end, and the small RNA matching them (B, C, D) Click here for file

Additional file 5 snoRNA derived small RNA. Predicted secondary structure of snoRNA, mgU6-77, and the small RNAs derived from this (indicated with a dashed box). Click here for file

Additional file 6 snRNA derived small RNA. Alignments of snRNA sequences and their derived small RNAs Click here for file

Additional file 7 Length distributions of small RNAs derived from protein coding genes. Histograms are shown in the same way to the additional file 2. Click here for file

Additional file 8 Predicted secondary structure of CEND1 proximal region. Predicted secondary structure of the proximal region (chr11:777439,777638) of the small RNAs located at the antisense of CEND1 gene. The region corresponding to the small RNA is indicated with red Click here for file

Additional file 9 Small RNA clusters overlapping bidirectional promoters. Small RNA clusters overlapping bidirectional promoters are listed with their genomic coordinates and associated rRNAs Click here for file

Additional file 10 Bidirectional promoters, small RNAs, and interstitial rRNA loci. Genomic view of small RNA generating loci, which are associated with small RNA, bidirectional promoters, and interstitial rRNAs. Click here for file

Additional file 11 Interstitial rRNA. "rrna.psl" is the raw result of blat query for the genome with rRNA sequences. "rrna_overlapping_smallRNA.txt" and "rrna_overlapping_cage.txt" list the rrna aligned regions overlapping with the small RNA and the CAGE, respectively. Click here for file

Additional file 12 Novel miRNA sequences. Hairpin sequences are described, where lower-case letters show the mature sequences. Click here for file

Additional file 13 Predicted secondary structures of the novel miRNA precursors. Predicted hairpin structures of the novel miRNAs are shown. The region corresponding to the mature miRNAs are indicated with red. Click here for file

Additional file 14 miRNAs derived from H19. Predicted hairpin structure of the miRNA (A), and genomic view of the loci. Click here for file

Additional file 15 5'- and 3'-end nucleotide frequencies of the tRNA derived small RNAs. The frequencies are plotted as bar plot. The left shows the frequencies computed based on for all reads, and the right shows one based on only the unique sequences. Click here for file

Additional file 16 Relationship between tRNAs originating the cleaved products and ones used as primers in LTR's reverse transcription. The Venn diagram shows if each of tRNA produces shorter or longer forms of the fragment, and involves in LTR reverse-transcription or not. Click here for file

Additional file 17 Counts of the tRNA derived small RNAs depending on the 3'-ends. Counts of the tRNA derived small RNAs per their length, with distinction of the 3'-ends (harboring CCA or not) Click here for file

Additional file 18 Small RNA sequences and their annotations. All of the unique sequences from our libraries are listed. DDBJ accession, internal id, count (or frequencies) of the sequence, length, annotations, RNA sequence, and clusters on the genome are described. Click here for file

Additional file 19 Genomic coordinates of the small RNA clusters. Genomic coordinates of the clusters are described in GFF format. Click here for file

Additional file 20 Counts of the small RNAs with alignment status. The number of small RNAs in each class is shown depending on the mapping methods: the mapping strategy adopted in this paper (A), and only the exact matches (B). Click here for file

Additional file 21 Northern probes. List of the probes used in the Northern blotting experiments. Click here for file
