# Supplementary material for: Hidden layers of human small RNAs
Source: BMC Genomics. 2008 Apr 10;9:157. doi: 10.1186/1471-2164-9-157 (PMC2359750; doi:10.1186/1471-2164-9-157)
Supplement: Additional file 1 — Schematic representation of the experimental protocols of small RNA library preparation. Ten steps in the protocols are shown. [file 1471-2164-9-157-S1.pdf]

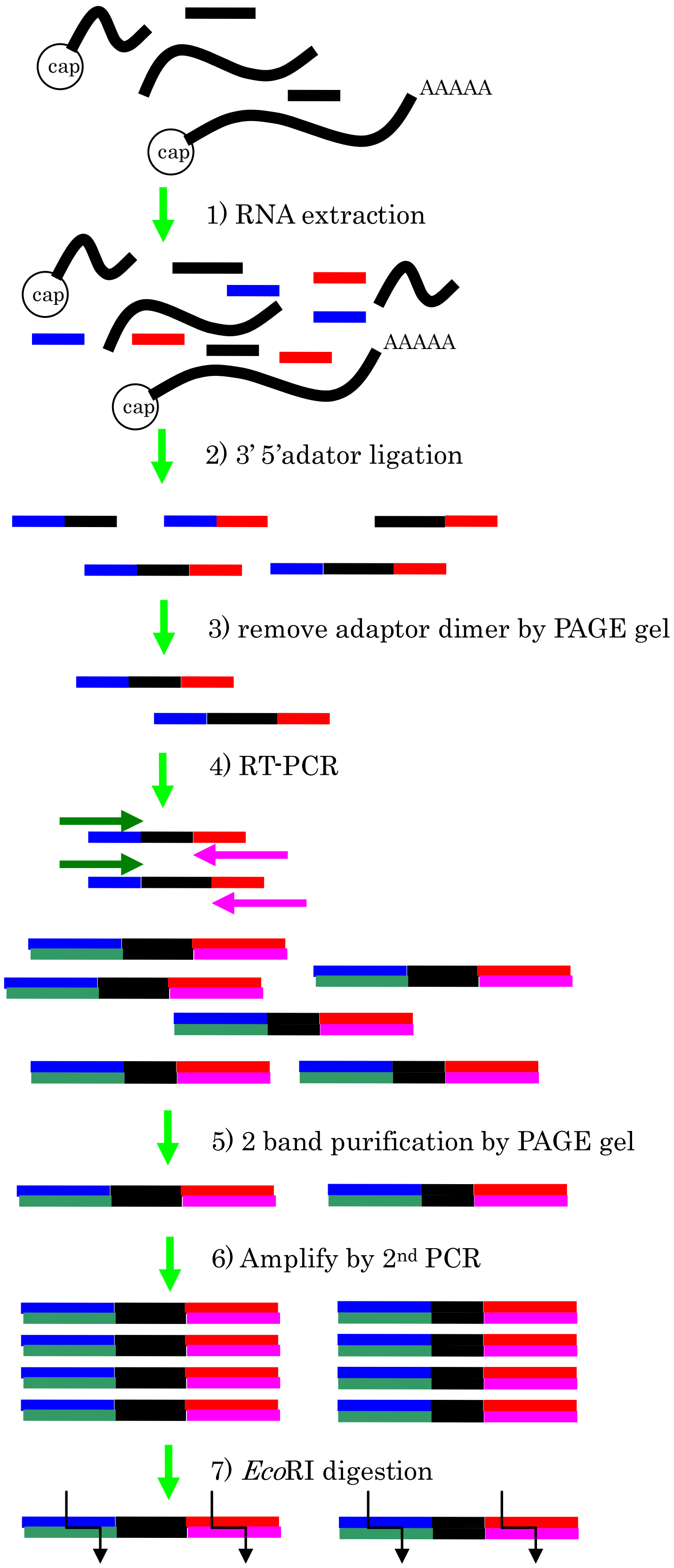
cap
AAAAA
cap
1) RNA extraction
cap
AAAAA
cap
2) 3' 5'adator ligation
3) remove adaptor dimer by PAGE gel
4) RT-PCR
5) 2 band purification by PAGE gel
6) Amplify by 2nd PCR
7) *Eco*RI digestion

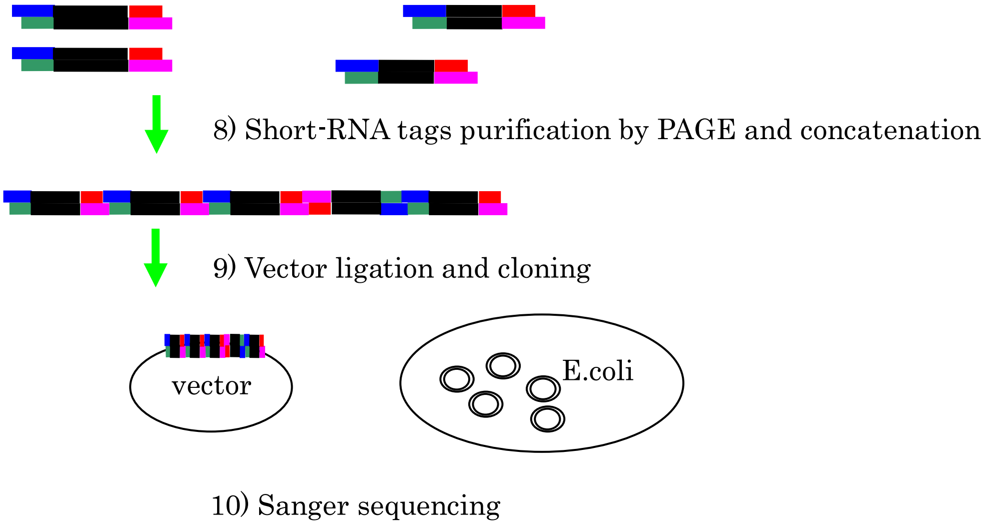

1) HepG2 total RNA are extracted by AGPC method minimizing the time in order to avoid random cleavage of RNA.
2) 3' and 5' RNA-DNA chimeric oligonucleotides are ligated to the short RNAs.
3) Removal of adaptor dimers by PAGE gel electrophoresis
4) Reverse transcriptase and 1st PCR cycle
5) The two main bands are separated by PAGE gel from non-specific products
6) 2nd PCR to amplify of DNA fragment
7) Rhort RNA tags are cut by using the restriction enzyme EcoRI
8) Short RNA tags are purification by PAGE gel and then concatenated
9) Concatemers are cloned into the p-ZeroII vector and transformed
10) sequence by using RISA system (Sanger based).
