# Supplementary figures and images for: Hidden layers of human small RNAs
Source: BMC Genomics. 2008 Apr 10;9:157. doi: 10.1186/1471-2164-9-157 (PMC2359750; doi:10.1186/1471-2164-9-157)

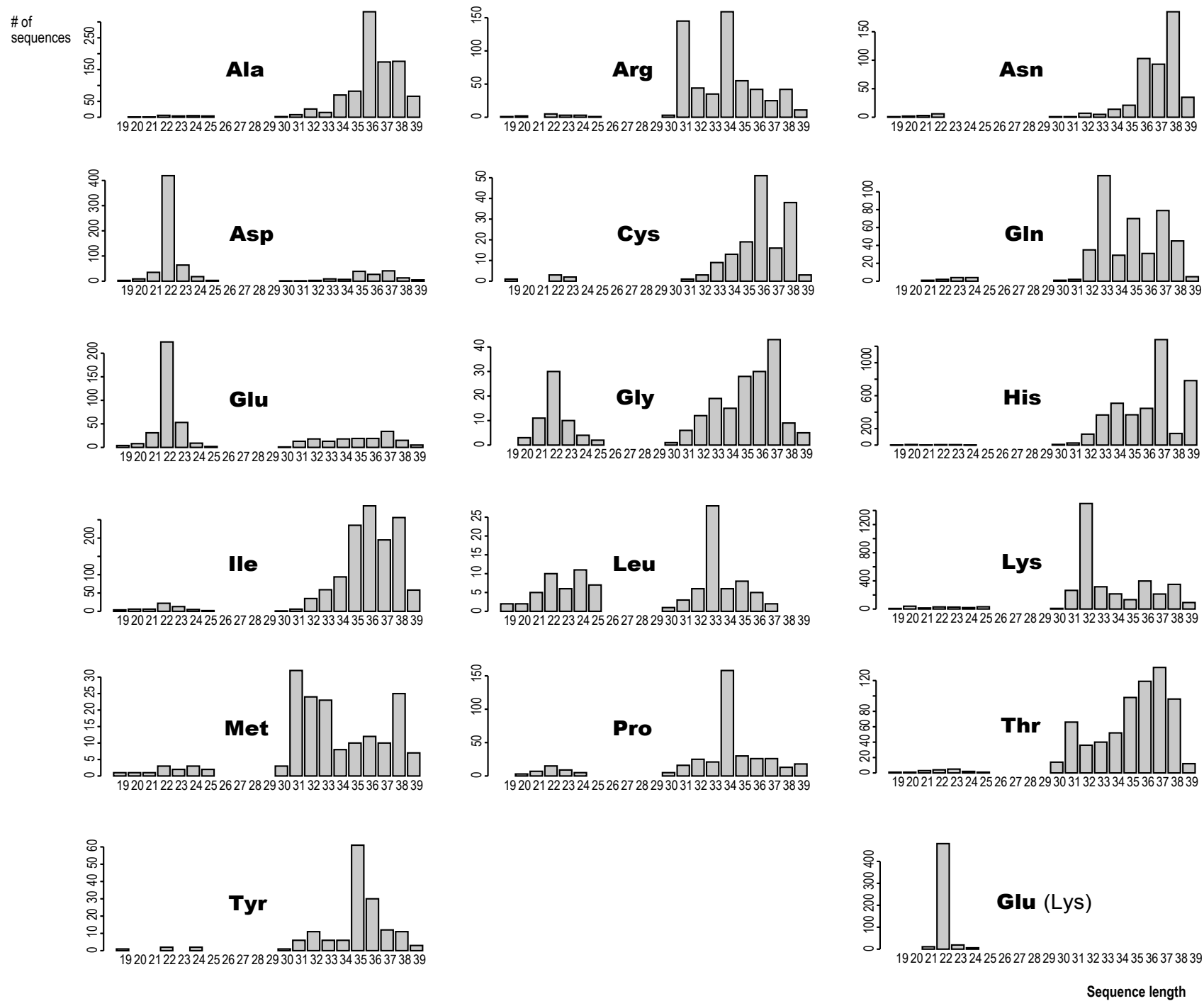

Supplement: Additional file 2 — Length distributions of tRNA derived smallRNAs per isoacceptor. The distributions are shown as histograms, where X and Y axes mean the length of the small RNAs and their frequencies, respectively. [file 1471-2164-9-157-S2.pdf]

# of  
sequences

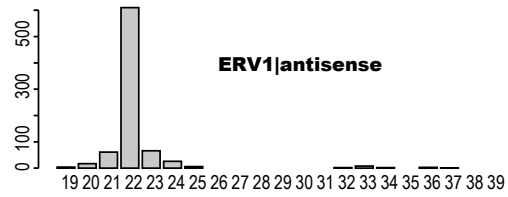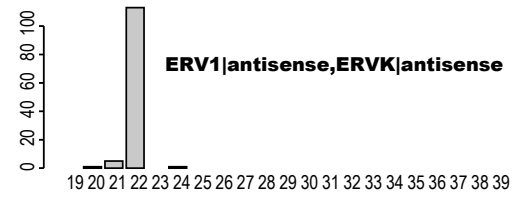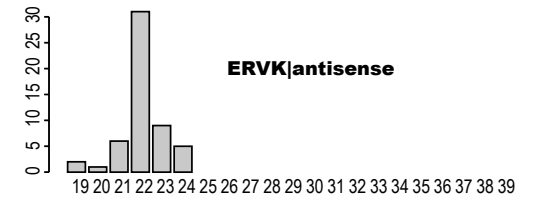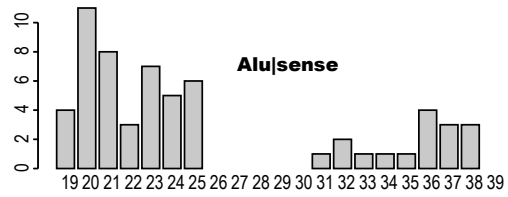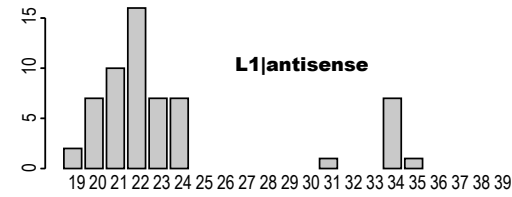

Sequence length

Supplement: Additional file 3 — Length distributions of repeat derived small RNAs per repeat classes and strands. Histograms are shown in the same way to the additional file 2. [file 1471-2164-9-157-S3.pdf]

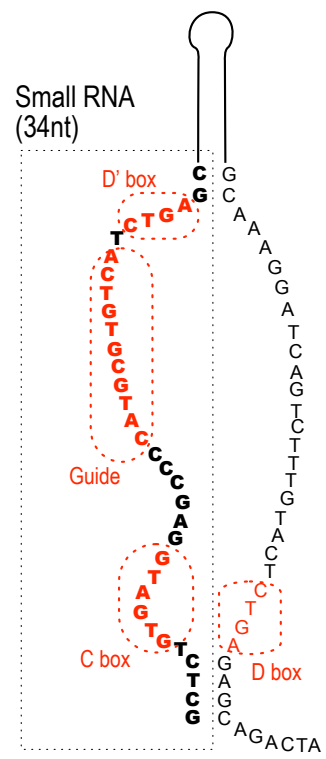

Supplement: Additional file 5 — snoRNA derived small RNA. Predicted secondary structure of snoRNA, mgU6-77, and the small RNAs derived from this (indicated with a dashed box). [file 1471-2164-9-157-S5.pdf]

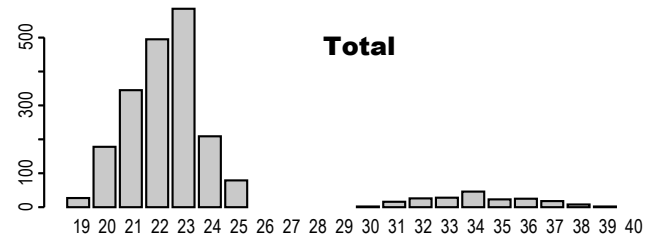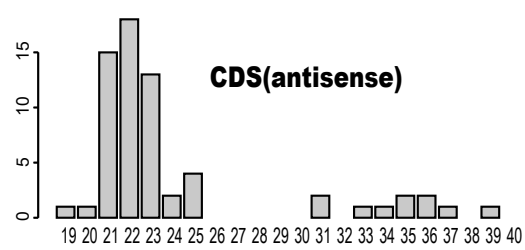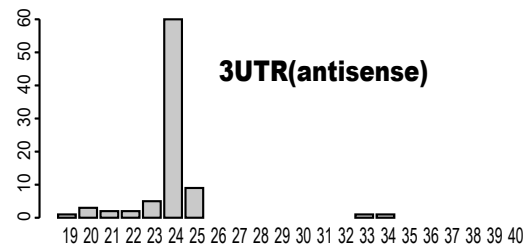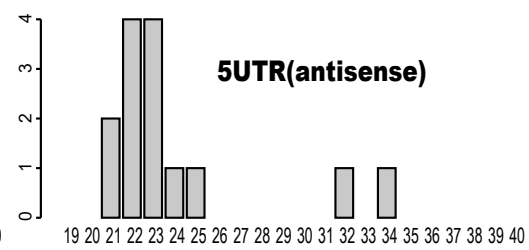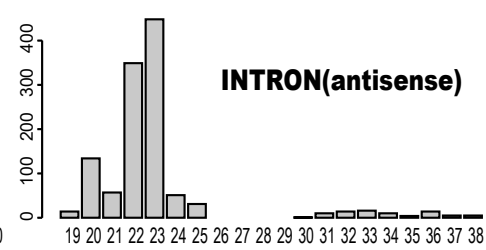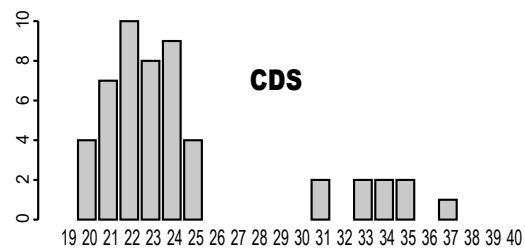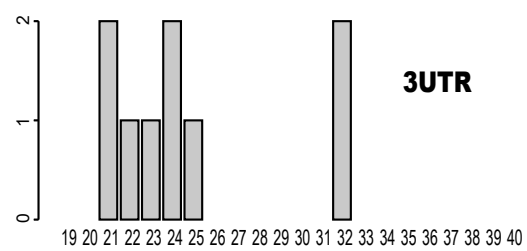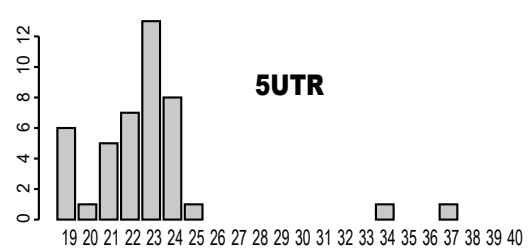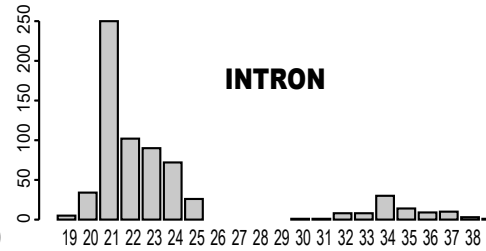

Supplement: Additional file 7 — Length distributions of small RNAs derived from protein coding genes. Histograms are shown in the same way to the additional file 2. [file 1471-2164-9-157-S7.pdf]

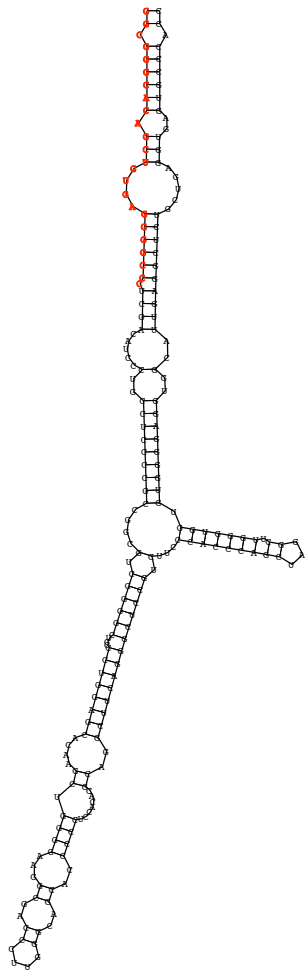

Supplement: Additional file 8 — Predicted secondary structure of CEND1 proximal region. Predicted secondary structure of the proximal region (chr11:777439,777638) of the small RNAs located at the antisense of CEND1 gene. The region corresponding to the small RNA is indicated with red [file 1471-2164-9-157-S8.pdf]

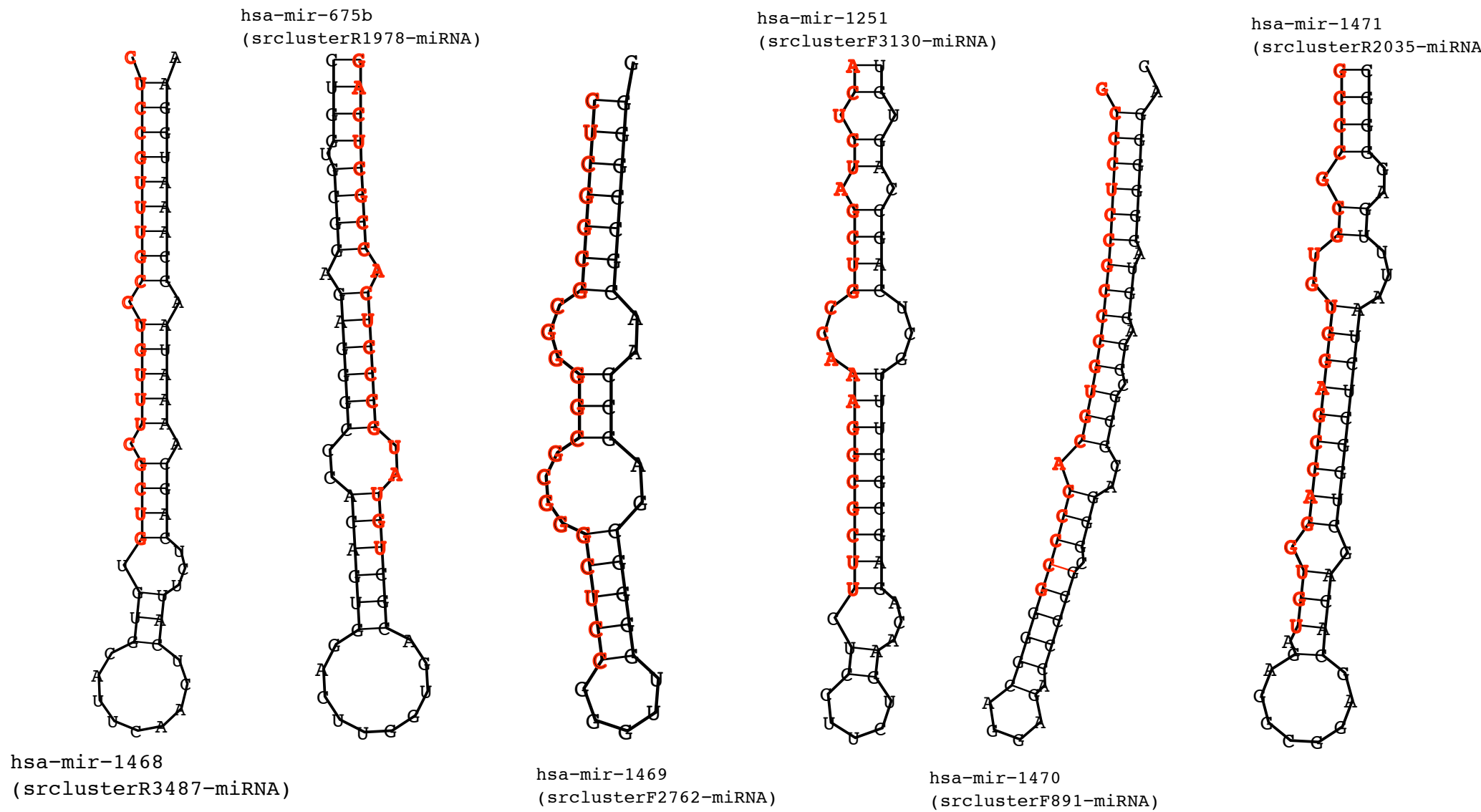

Figure S14. Predicted secondary structures of the novel miRNAs

Supplement: Additional file 13 — Predicted secondary structures of the novel miRNA precursors. Predicted hairpin structures of the novel miRNAs are shown. The region corresponding to the mature miRNAs are indicated with red. [file 1471-2164-9-157-S13.pdf]

(A)

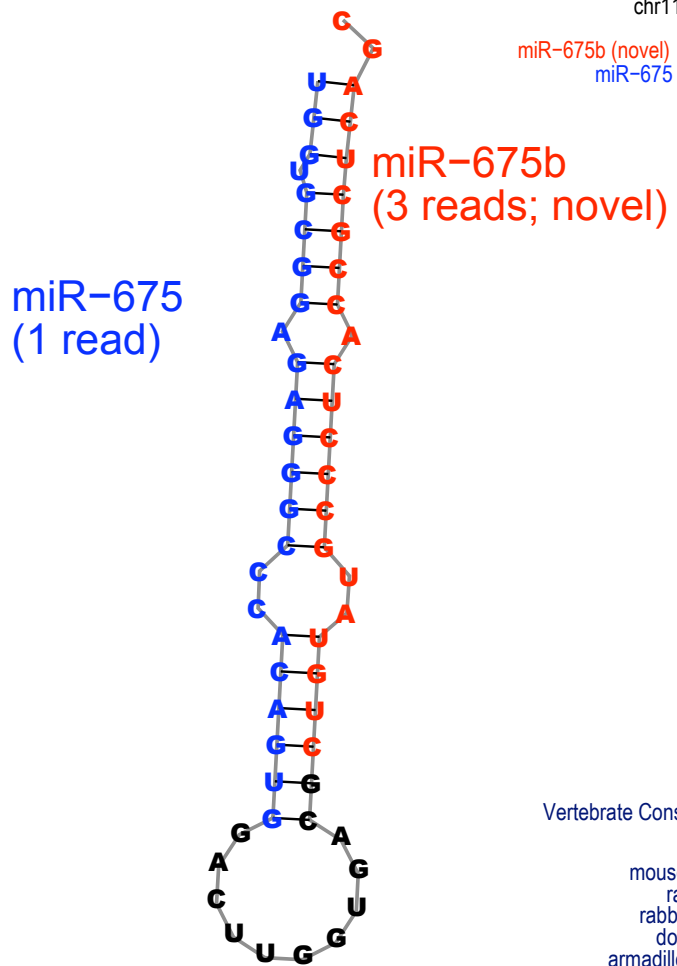

(B)

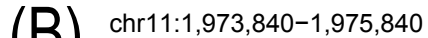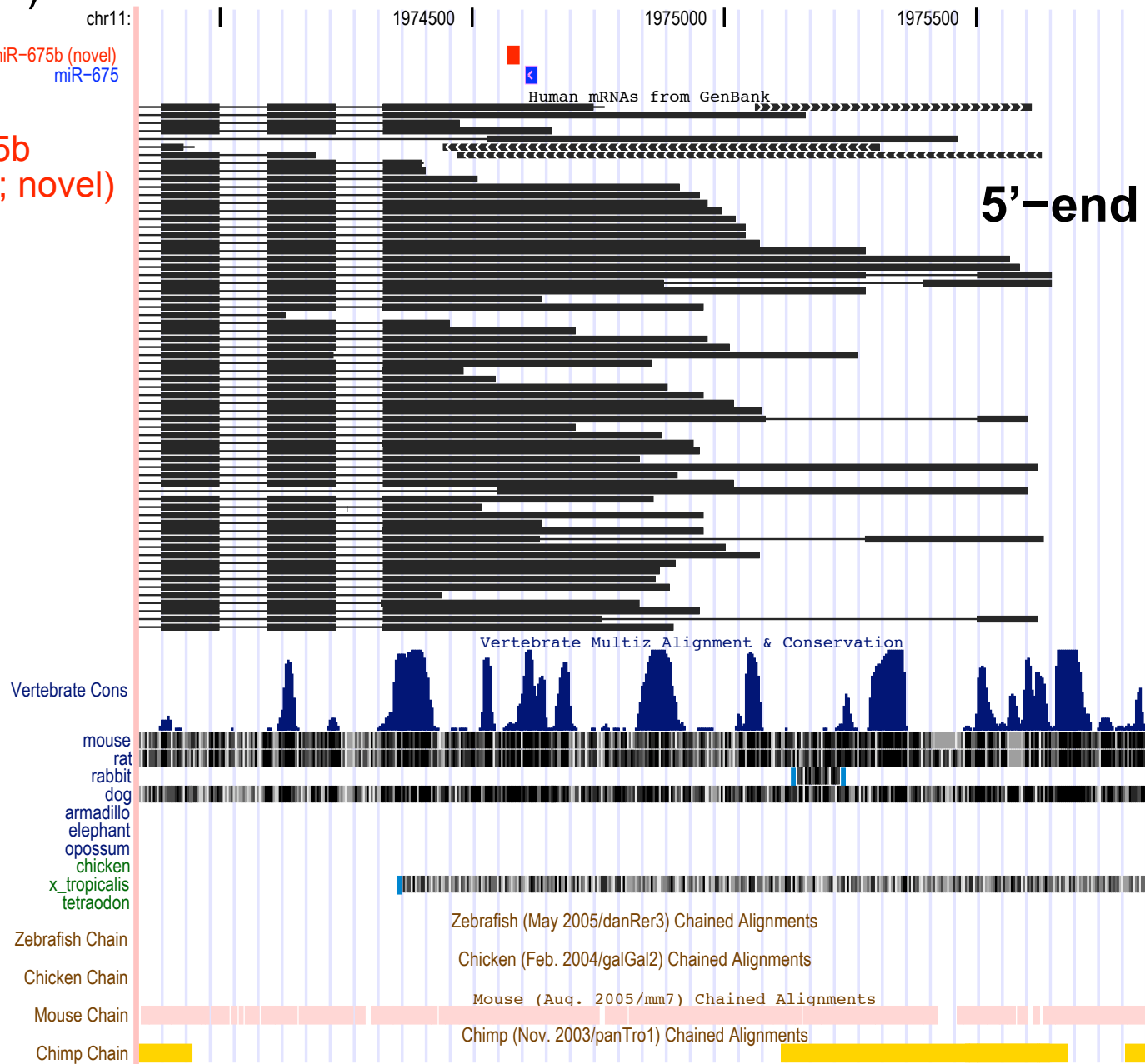

Supplement: Additional file 14 — miRNAs derived from H19. Predicted hairpin structure of the miRNA (A), and genomic view of the loci. [file 1471-2164-9-157-S14.pdf]

all reads

5'-end

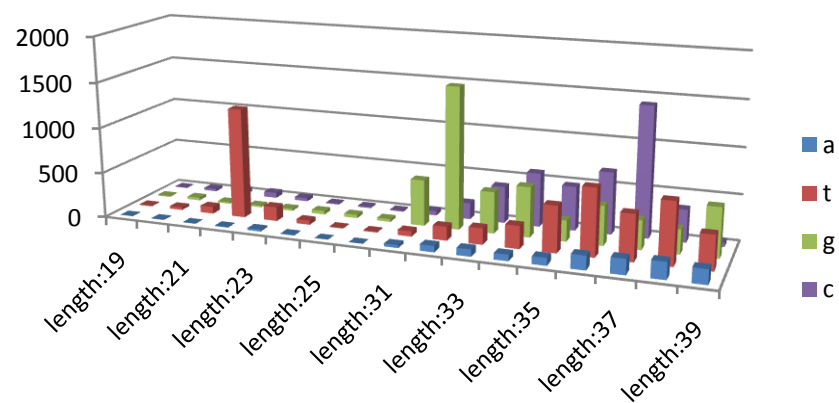

unique (non-redundant) sequences

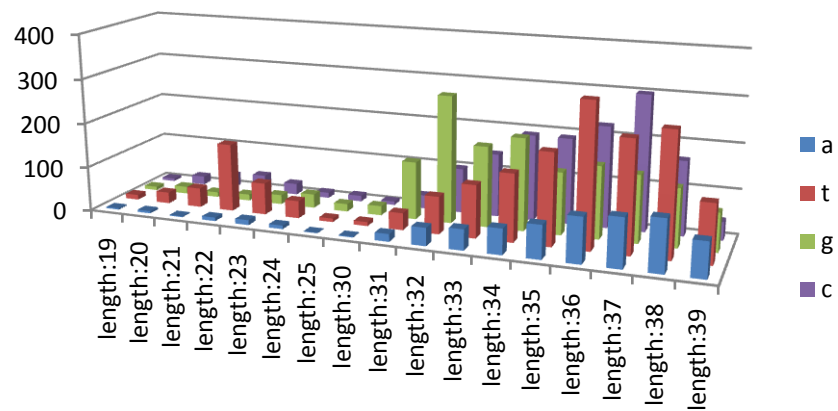

3'-end

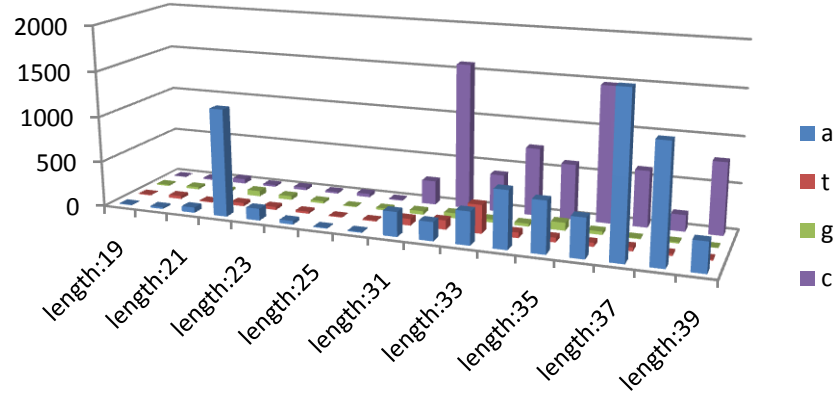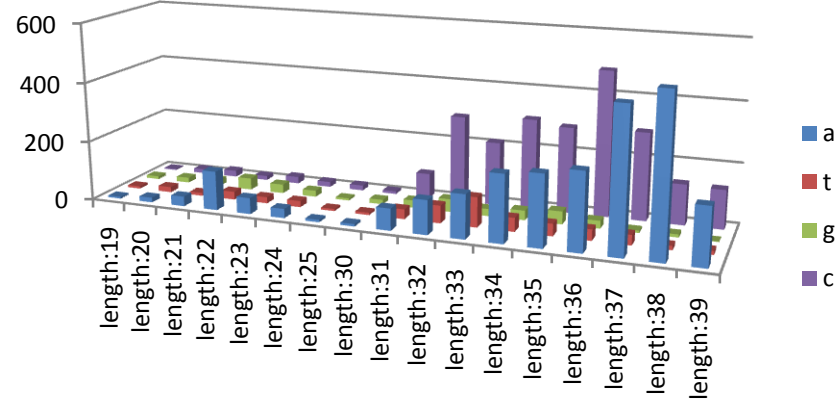

Supplement: Additional file 15 — 5'- and 3'-end nucleotide frequencies of the tRNA derived small RNAs. The frequencies are plotted as bar plot. The left shows the frequencies computed based on for all reads, and the right shows one based on only the unique sequences. [file 1471-2164-9-157-S15.pdf]

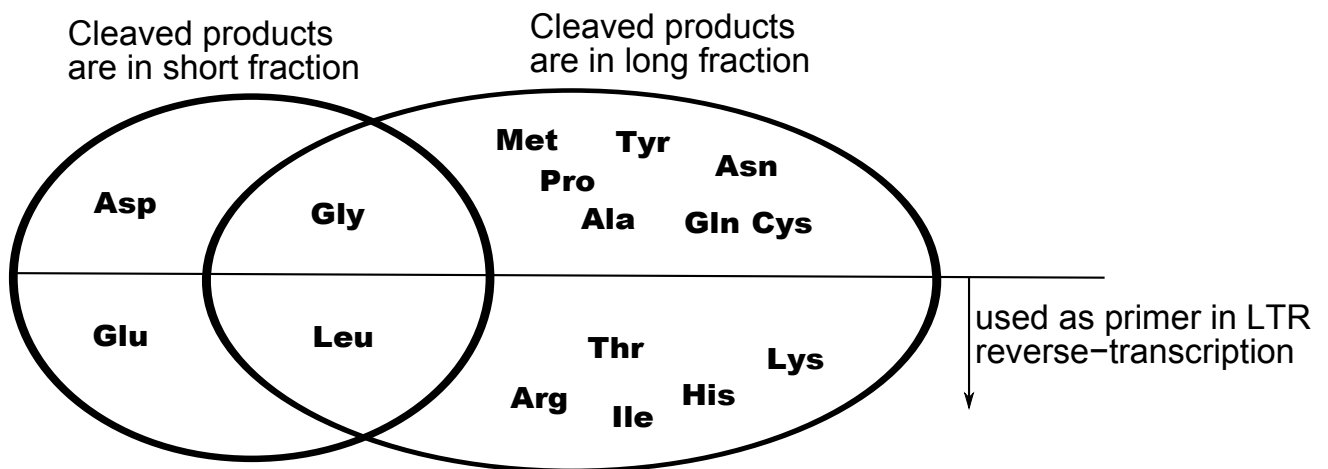

Figure S10. Categories of the tRNA isoacceptor.

Supplement: Additional file 16 — Relationship between tRNAs originating the cleaved products and ones used as primers in LTR's reverse transcription. The Venn diagram shows if each of tRNA produces shorter or longer forms of the fragment, and involves in LTR reverse-transcription or not. [file 1471-2164-9-157-S16.pdf]

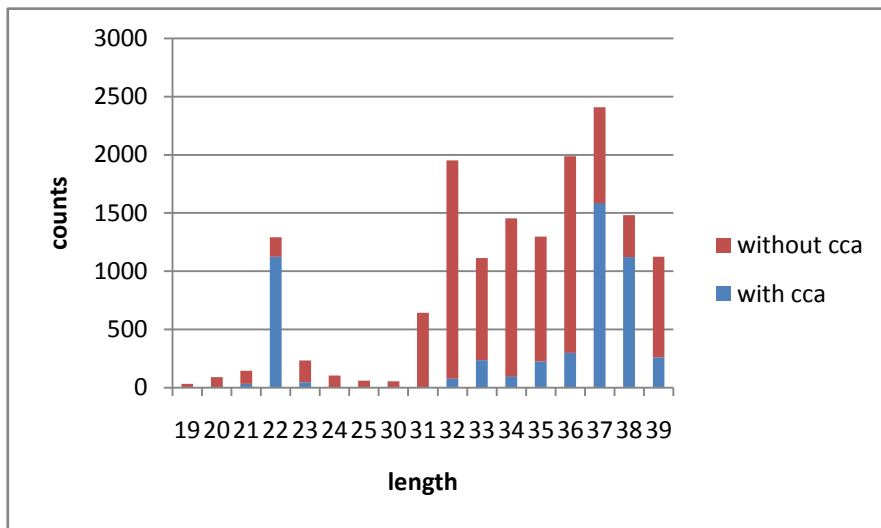

Supplement: Additional file 17 — Counts of the tRNA derived small RNAs depending on the 3'-ends. Counts of the tRNA derived small RNAs per their length, with distinction of the 3'-ends (harboring CCA or not) [file 1471-2164-9-157-S17.pdf]

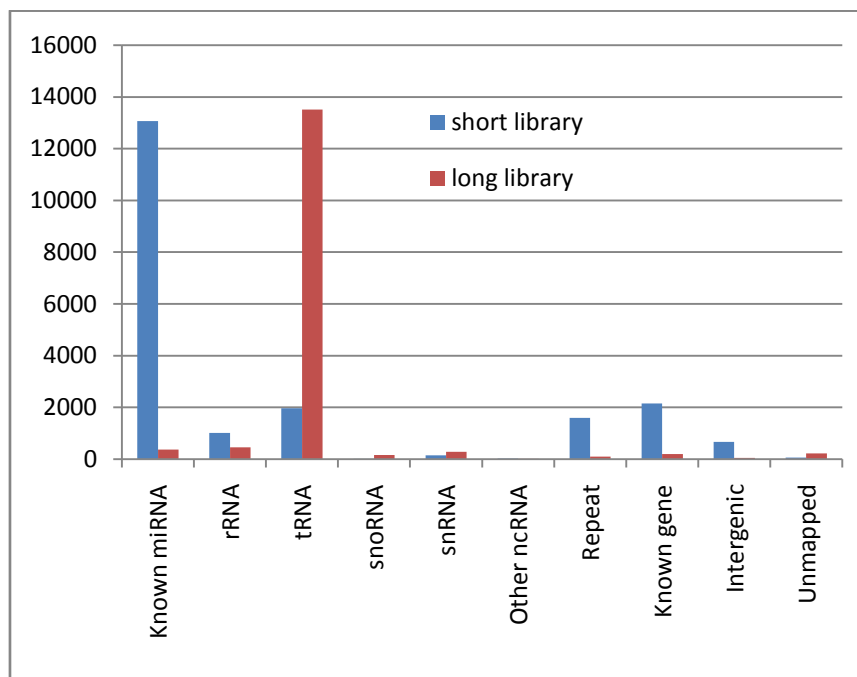

(A) Counts of the all reads

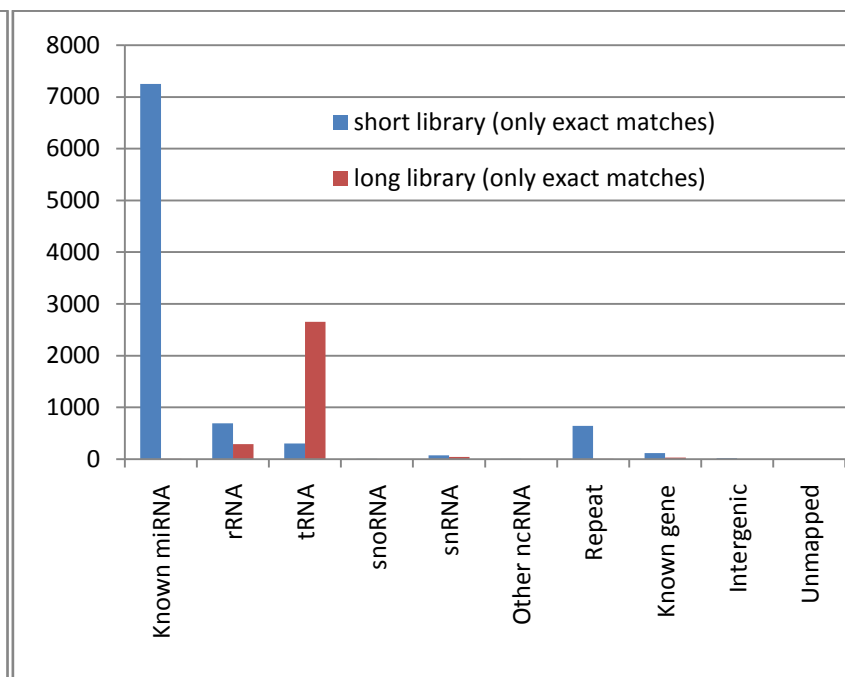

(B) counts of the reads which are perfectly aligned with the genome

Supplement: Additional file 20 — Counts of the small RNAs with alignment status. The number of small RNAs in each class is shown depending on the mapping methods: the mapping strategy adopted in this paper (A), and only the exact matches (B). [file 1471-2164-9-157-S20.pdf]
