# Supplementary material for: Hidden layers of human small RNAs
Source: BMC Genomics. 2008 Apr 10;9:157. doi: 10.1186/1471-2164-9-157 (PMC2359750; doi:10.1186/1471-2164-9-157)
Supplement: Additional file 4 — Relationship between tRNA and LTR. Schematic structure of tRNA priming of LTR reverse transcription (A), and alignment of LTR's primer binding site (PBS), tRNA 3'-end, and the small RNA matching them (B, C, D) [file 1471-2164-9-157-S4.pdf]

(A) LTR 5'−end structure

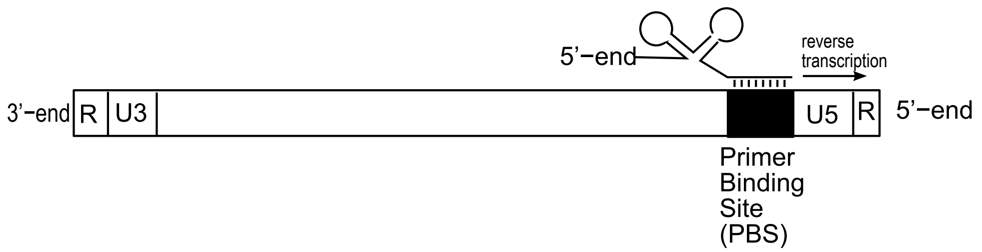

(B) PBS of Harlequin (ERV1)

```
3'-AGCAAAGGGCCAGTCCCTTGGT-5'   PBS of Harlequin
                               (chr1:168,301,725-168,301,747)
   ||||||||||||||||||||||
5'-TCGTTTCCCGGTCAGGGAACCA-3'   Our small RNA sequence
                               (zza22501a16t3.scf|369|390, n=499)
   ||| |||||||||||||||
5'-TCGATTCCCGGTCAGGGAA-3'      3'-end of tRNA
      ^                        (chr1.trna109-GluCTC)
     57th
```

(C) PBS of HERVK9 (ERVK), or MER61A(ERV1)

```
3'-AGCTCGGGGTGCAACCCGCGGT-5'   PBS of
                                   HERVK9      (chr5:51,636,598-51,636,619)
   ||| ||||||||||||||||||       or MER61A-int (chr6:76,792,740-76,792,761)
5'-TCGTGCCCCACGTTGGGCGCCA-3'   Our small RNA sequence
                               (zza22501g02t3.scf|184|205, n=70)
   ||| |||||||||||||||
5'-TCGAGCCCCACGTTGGGCG-3'      3'-end of tRNA
      ^                        (chr16.trna10-LysCTT)
     58th
```

(D) PBS of HERVK3 (ERVK) , or LTR3B (ERVK)

```
3'-AGTGCAGGGACAAGCCCGCGGT-5'   PBS of HERVK3, or LTR3B
                               (chr19:62,716,223-62,716,244)
   ||| ||||||||||||||||||
5'-TCAGGTCCCTGTTCGGGCGCCA-3'   Our small RNA sequence
                               (zza22504m18t3.scf|384|405, n=18)
   ||| |||||||||||||||
5'-TCAAGTCCCTGTTCGGGCG-3'      3'-end of tRNA
      ^                        (chr11.trna5-LysTTT)
     58th
```
