# Supplementary material for: Hidden layers of human small RNAs
Source: BMC Genomics. 2008 Apr 10;9:157. doi: 10.1186/1471-2164-9-157 (PMC2359750; doi:10.1186/1471-2164-9-157)
Supplement: Additional file 6 — snRNA derived small RNA. Alignments of snRNA sequences and their derived small RNAs [file 1471-2164-9-157-S6.pdf]

U1

```
AUACUUACCU GGCAGGGGAG AUACCAUGAU CACGAAGGUG GUUUUCCCAG 50

GGCGAGGCUU AUCCAUUGCA CUCCGGAUGU GCUGACCCCU GCGAUUUCCC 100

CAAAUGUGGG AAACUCGACU GCAUAAUUUG UGGUAGUGGG GGACUGCGUU 150
                                    ||||||| ||||||||||
                                    TAGTGGG GGACTGCGTT
CGCGCUUUCC CCUG
|||||| ||| ||||
CGCGCTCTCC CCTG
```

U4

```
AGCUUUGCGC AGUGGCAGUA UCGUAGCCAA UGAGGUUUAU CCGAGGCGCG 50

AUUAUUGCUA AUUGAAAACU UUUCCCAAUA CCCCGCCGUG ACGACUUGCA 100

AUAUAGUCGG CAUUGGCAAU UUUUGACAGU CUCUACGGAG
                      |||||||||| ||||||||||
                      TTTTGACAGT CTCTACGGAGACTG
```

U5

```
ATACTCTGGT TTCTCTTCAG ATCGCATAAA TCTTTCGCCT TTTACTAAAG 50

ATTTCCGTGG AGAGGAACAA CTCTGAGTCT TAACCCAATT TTTTGAGGCC 100
                                               |||||||
                                               TGAGGCC
TTGCTTTGGC AAGGCTA
||| || ||| |||||||
TTGTTTCGGC AAGGCTA
```
