# Supplementary material for: Hidden layers of human small RNAs
Source: BMC Genomics. 2008 Apr 10;9:157. doi: 10.1186/1471-2164-9-157 (PMC2359750; doi:10.1186/1471-2164-9-157)
Supplement: Additional file 9 — Small RNA clusters overlapping bidirectional promoters. Small RNA clusters overlapping bidirectional promoters are listed with their genomic coordinates and associated rRNAs [file 1471-2164-9-157-S9.pdf]

No	genomic coordinates	aligned rRNA	
1	chr8:70764954		
9	chr8:70765150	28S_rRNA	
2	chr1:91564913	28S_rRNA	
3	chrX:108103880		
12	chrX:108103958	28S_rRNA	
4	chrY:10629479	28S_rRNA	
5	chr5:149662199	28S_rRNA	Just only 24bp
6	chr16:33871232		
8	chr16:33871421	5.8S_rRNA	
7	chr2:132872372	28S_rRNA	
10	chr5:71182526	28S_rRNA	
11	chr2:132847183	18S_rRNA	
