# Supplementary material for: Hidden layers of human small RNAs
Source: BMC Genomics. 2008 Apr 10;9:157. doi: 10.1186/1471-2164-9-157 (PMC2359750; doi:10.1186/1471-2164-9-157)
Supplement: Additional file 10 — Bidirectional promoters, small RNAs, and interstitial rRNA loci. Genomic view of small RNA generating loci, which are associated with small RNA, bidirectional promoters, and interstitial rRNAs. [file 1471-2164-9-157-S10.pdf]

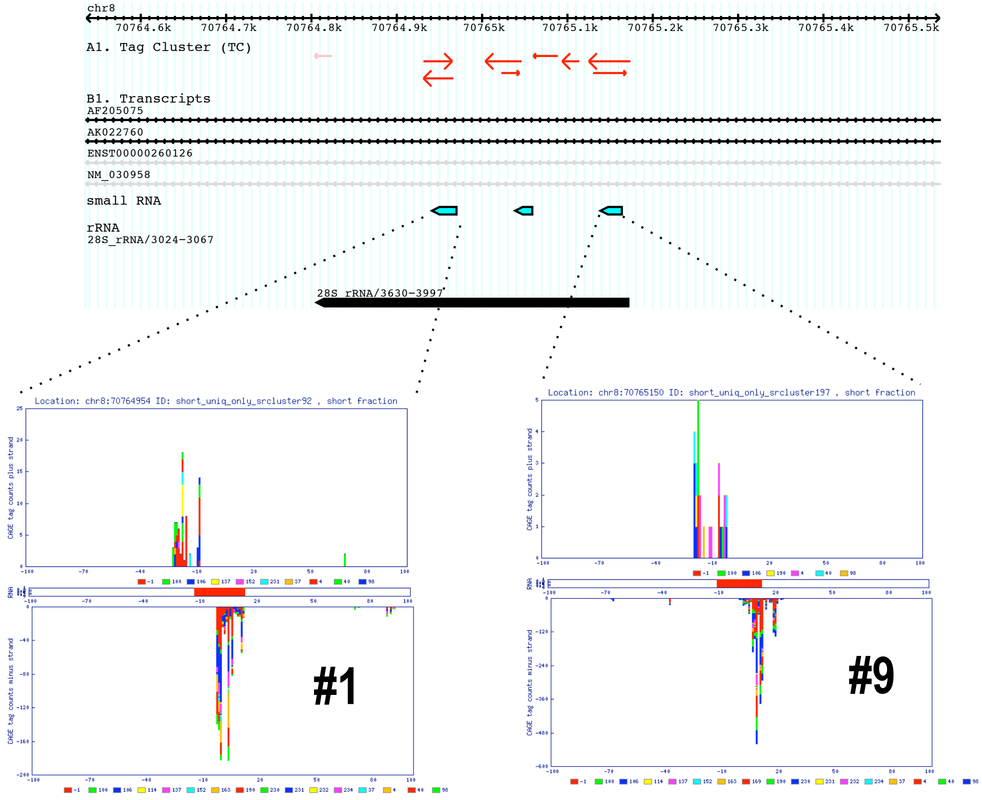

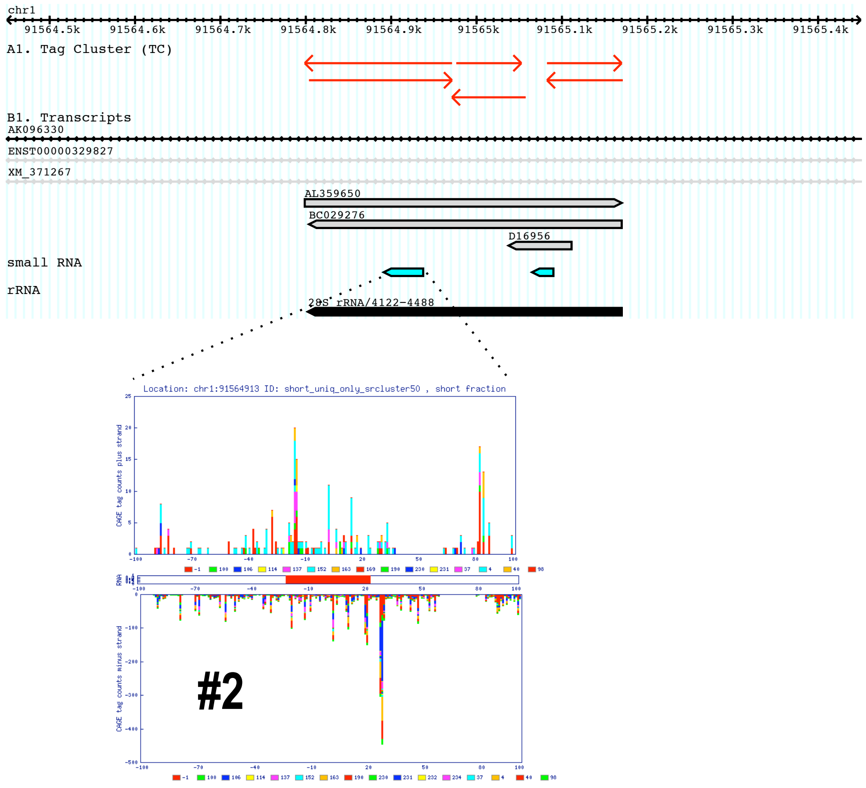

Different colors in the histograms represent CAGE tags deriving from different tissues. Tissue name for each ID are described here:

ID	Tissue name
-1	UNDEFINED_TISSUE_TYPE
100	large intestine
106	liver
114	mammary gland
137	pancreas
152	rectum
163	small intestine
169	stomach
190	urinary bladder
230	ureter
231	renal artery
232	prostate gland
234	epididymis
37	cecum
4	adipose
40	cerebrum
98	kidney

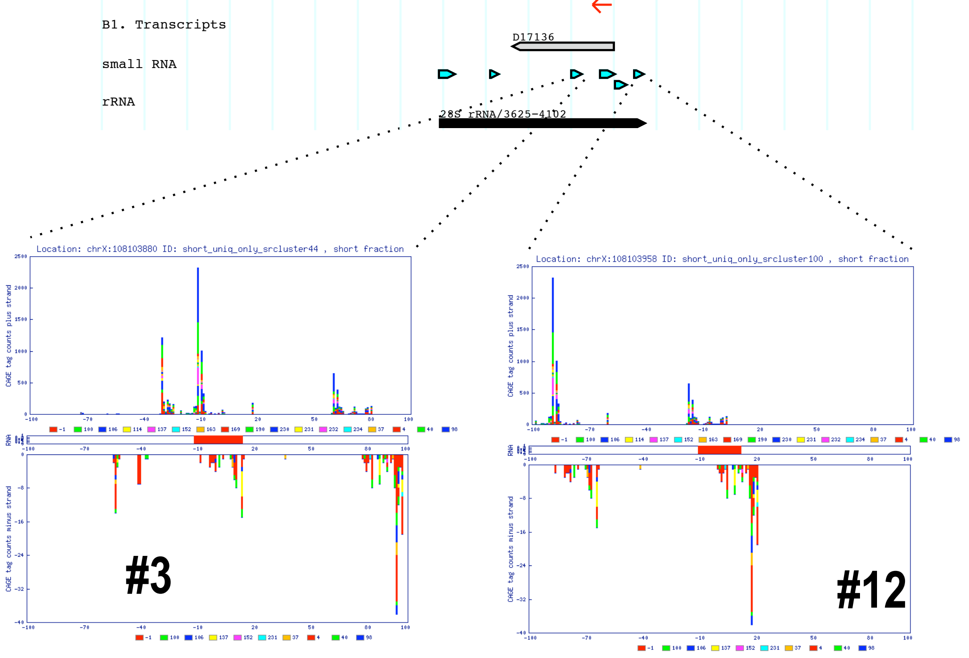

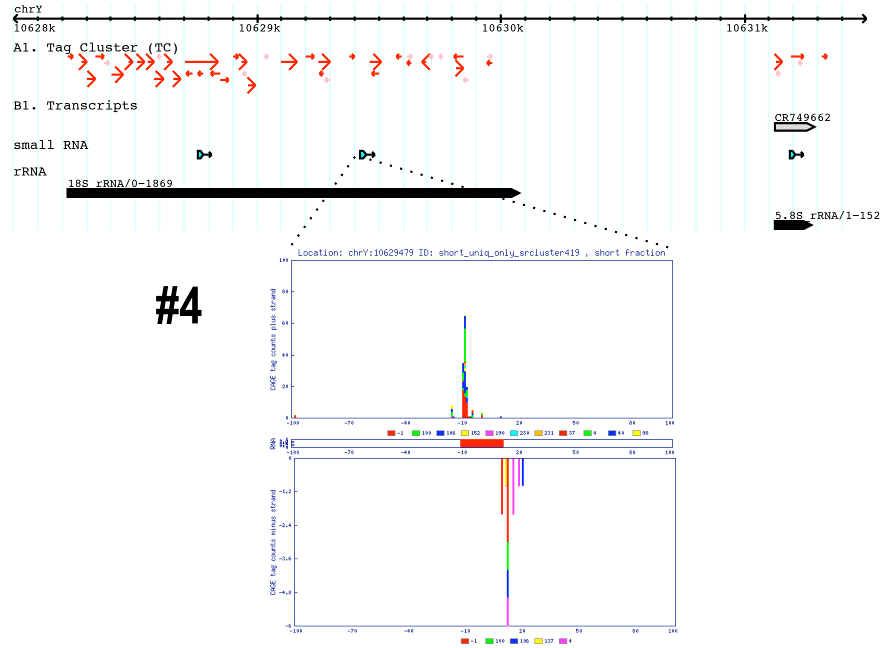

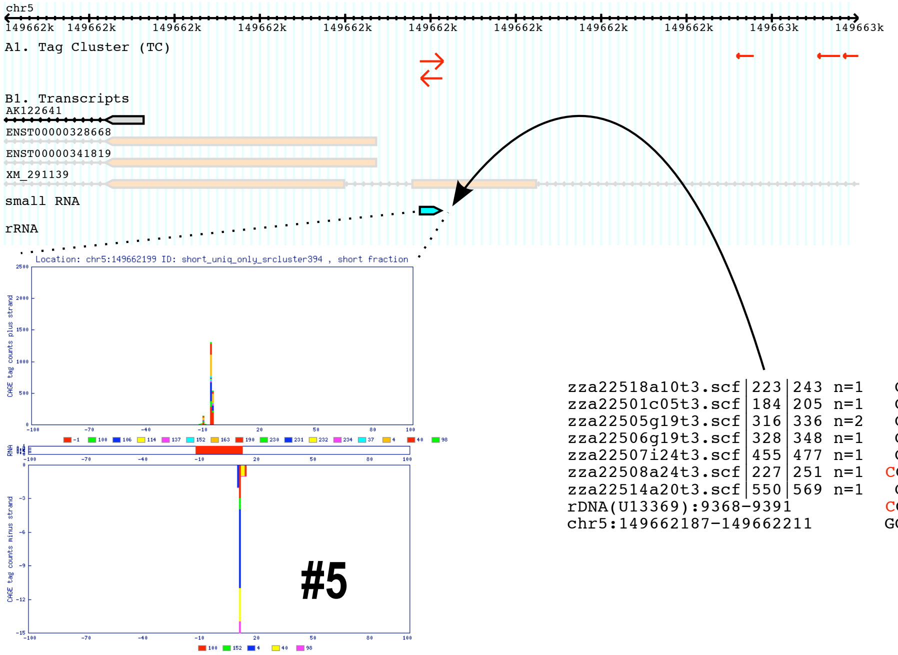

zza22518a10t3.scf	223	243	n=1	CGCCAAGGGCCCACCACCGGC
zza22501c05t3.scf	184	205	n=1	CGCCGAGGGCGCACCACCGGCC
zza22505g19t3.scf	316	336	n=2	CGCCGAGGGCGCACCACCGGC
zza22506g19t3.scf	328	348	n=1	CGCCGAGTGCGCACCACCGGC
zza22507i24t3.scf	455	477	n=1	CGCCGAGGGCGCACCACCGGCTC
zza22508a24t3.scf	227	251	n=1	CCGCCGAGGGCGCACCACCGGCCCG
zza22514a20t3.scf	550	569	n=1	CGCCGAGGGCGCACCATCGG
rDNA(U13369):9368-9391				CCGCCGAGGGCGCACCACCGGCCCG
chr5:149662187-149662211				GCGCCCAGGGCGCACCACCGGCCCG

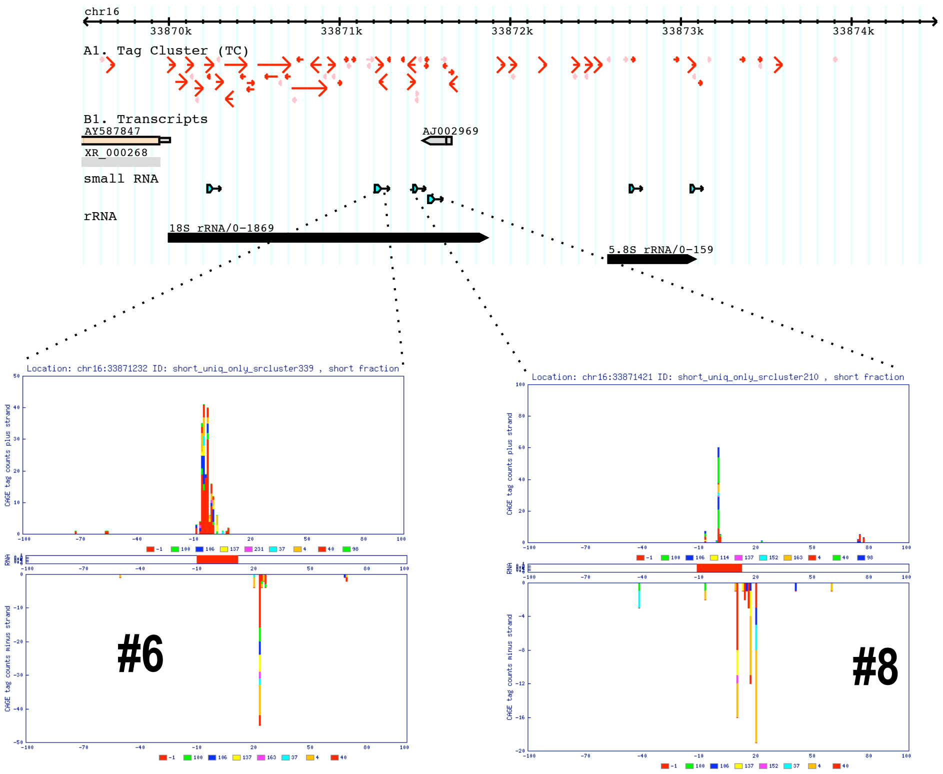
chr16
33870k
33871k
33872k
33873k
33874k
A1. Tag Cluster (TC)
B1. Transcripts
AY587847
AJ002969
XR_000268
small RNA
rRNA
18S rRNA/0-1869
5.8S rRNA/0-159
Location: chr16:33871232 ID: short_uniq_only_srcluster339 , short fraction
Location: chr16:33871421 ID: short_uniq_only_srcluster210 , short fraction
#6
#8

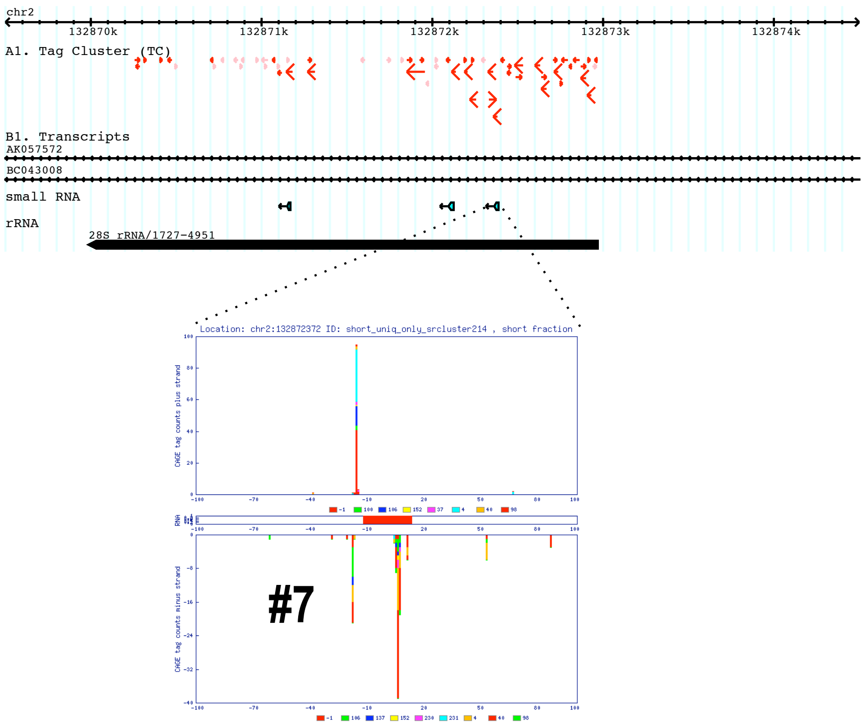
chr2
132870k
132871k
132872k
132873k
132874k
A1. Tag Cluster (TC)
B1. Transcripts
AK057572
BC043008
small RNA
rRNA
28S rRNA/1727-4951
Location: chr2:132872372 ID: short_uniq_only_srcluster214 , short fraction
#7

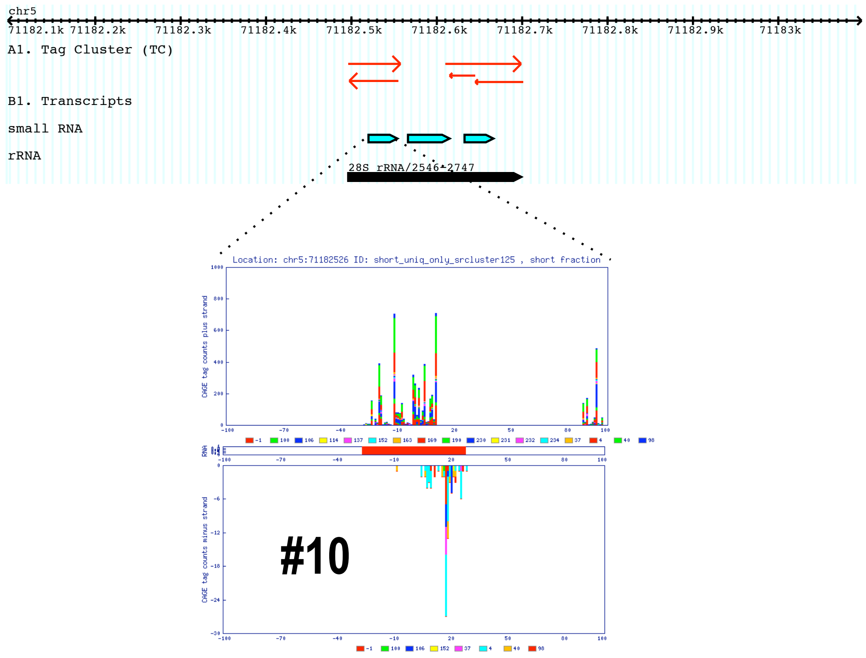
chr5
71182.1k 71182.2k 71182.3k 71182.4k 71182.5k 71182.6k 71182.7k 71182.8k 71182.9k 71183k
A1. Tag Cluster (TC)
B1. Transcripts
small RNA
rRNA
28S_rRNA/2546-2747
Location: chr5:71182526 ID: short_uniq_only_srccluster125 , short fraction
CAGE tag counts plus strand
CAGE tag counts minus strand
#10

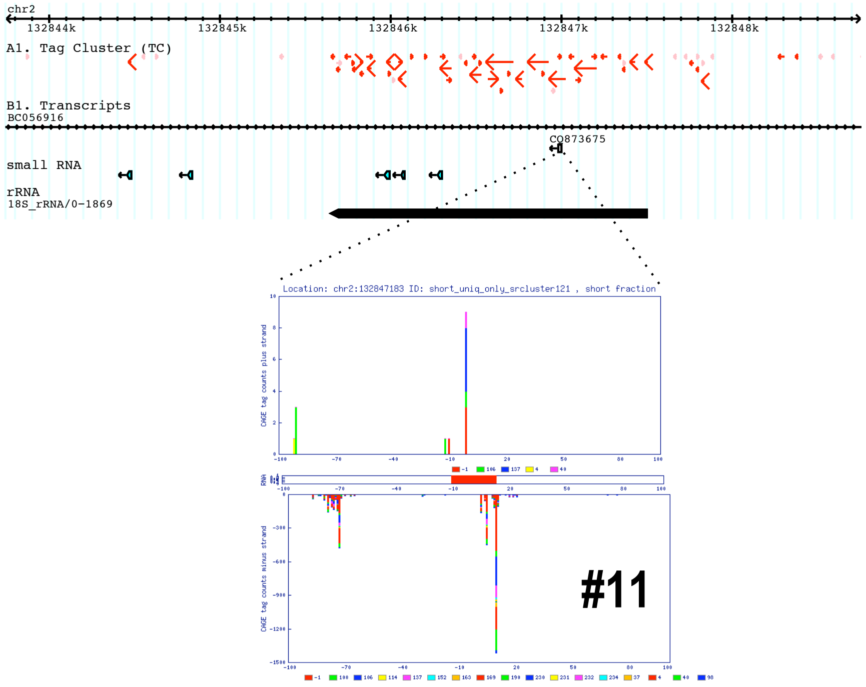
chr2
132844k 132845k 132846k 132847k 132848k
A1. Tag Cluster (TC)
B1. Transcripts
BC056916
CO873675
small RNA
rRNA
18S_rRNA/0-1869
Location: chr2:132847183 ID: short_uniq_only_srccluster121 , short fraction
CAGE tag counts plus strand
CAGE tag counts minus strand
#11
