# Supplementary material for: Hidden layers of human small RNAs
Source: BMC Genomics. 2008 Apr 10;9:157. doi: 10.1186/1471-2164-9-157 (PMC2359750; doi:10.1186/1471-2164-9-157)
Supplement: Additional file 21 — Northern probes. List of the probes used in the Northern blotting experiments. [file 1471-2164-9-157-S21.pdf]

tRNA	end	length	sequence
GluCTC	3'	22nt	TCGATTCCCGGTCAGGGAACCA
AspGTC	3'	22nt	TCGATTCCCCGACGGGGAGCCA
HisGTG	3'	37nt	CGCAGCAACCTCGGTTCGTATCCGAGTCACGGCACCA
IleTAT	3'	35nt	TGCCGAGGTTGTGAGTTCGGGCCTCACCTGGAGCA
IleTAT	5'	38nt	GCTCCAGTGGCGCAATCGGTTAGCGCGCGGTACTTATA
LysCTT	5'	32nt	GCCCGGCTAGCTCAGTCGGTAGAGCATGGGAC
AsnGTT	3'	38nt	CCGAAAGGTTGGTGGTTCGGGCCCACCCAGGGACGCCA
